# Risk of Developing Alzheimer’s Disease and Related Dementias in Association with Cardiovascular Disease, Stroke, Hypertension, and Diabetes in a Large Cohort of Women with Breast Cancer and with up to 26 Years of Follow-Up

**DOI:** 10.3233/JAD-215657

**Published:** 2022

**Authors:** Xianglin L. Du, Lulu Song, Paul E. Schulz, Hua Xu, Wenyaw Chan

**Affiliations:** a Department of Epidemiology, Human Genetics and Environmental Sciences, School of Public Health, The University of Texas Health Science Center at Houston, Houston, TX, USA; b Department of Neurology, The University of Texas Health Science Center at Houston, Houston, TX, USA; c School of Biomedical Informatics, The University of Texas Health Science Center at Houston, Houston, TX, USA; d Department of Biostatistics and Data Science, School of Public Health, The University of Texas Health Science Center at Houston, Houston, TX, USA

**Keywords:** Alzheimer’s disease, breast cancer, dementias, medicare, vascular diseases

## Abstract

**Background:**

No study on the long-term incidence of Alzheimer’s disease (AD) and related dementias (ADRD) has been reported in women with breast cancer by vascular diseases.

**Objective:**

To determine the risk of ADRD in association with cardiovascular diseases (CVD), stroke, hypertension, and diabetes in women with breast cancer.

**Methods:**

Study identified 246,686 women diagnosed with breast cancer at age ≥65 years in 1991–2015 from the Surveillance, Epidemiology, and End Results (SEER)-Medicare linked database. Women were free of ADRD at the time of cancer diagnosis and followed from 1991 to 2016.

**Results:**

Cumulative incidence of AD over 26 years of follow-up varied from 10.7% to 13.6% by CVD, stroke, hypertension, and diabetes. Cumulative incidence of ADRD was higher in those with CVD (40.75%) versus no-CVD (31.32%), stroke (40.24%) versus no-stroke (31.34%), hypertension (33.06%) versus no-hypertension (30.47%), and diabetes (33.38%) versus no-diabetes (31.77%). After adjusting for confounders, those with CVD (hazard ratio:1.30, 95%CI: 1.27–1.33), stroke (1.50,1.47–1.54), hypertension (1.08,1.06–1.09), and diabetes (1.26,1.24–1.29) had significantly higher risks of developing ADRD. Women aged 80–84, and ≥85 had 5- and 7-fold higher risks of AD than those aged 65–69. As compared to white women, black women had a significantly higher risk of AD (1.21, 1.16–1.27), whereas Asians/Pacific-Islanders had a significantly lower risk of AD (0.77, 0.71–0.83).

**Conclusion:**

In women with breast cancer, CVD, stroke, hypertension, and diabetes were associated with a significantly higher risk of developing any ADRD combined. The risk of ADRD was higher in black women and lower in Asian/Pacific-Islanders than white women.

## INTRODUCTION

The prevalence of Alzheimer’s disease (AD) and related dementias (ADRDs) has been increasing over the past few decades [1–6] and is projected to be doubled by 2025 and tripled by 2050 [1–5]. The global number of individuals who live with dementia increased from 20.2 million (95% uncertainty interval: 17·4–23·5) in 1990 to 43·8 million (37·8–51·0) in 2016 [6]. In the United States, it is estimated that 5.8 million Americans of all ages live with AD in 2019, of which 5.6 million people (97%) are age ≥ 65 and 81% are age ≥ 75 [1–4]. AD is the 6th leading cause of death in the United States [1–3] and is still incurable although much progress has been made [1–3].

Etiologies for ADRD remained largely unknown, but they have been linked to a number of risk, history of psychiatric disorders, head trauma, genetic factors, and vascular diseases or related risk factors such as cardiovascular diseases (CVD), stroke, hypertension, and diabetes [1–11]. Numerous studies have documented that regular physical exercise and good management of cardiovascular risk factors (such as diabetes and hypertension) are associated with a reduced risk of cognitive decline and may be associated with a reduced risk for dementia [1–3, 9].

To the best of our knowledge, no study on the long-term incidence of ADRD has been reported in older women with breast cancer amongst Medicare beneficiaries. Because of early detection and significant improvements in treatment for breast cancer, women diagnosed with breast cancer now have higher survival rates. It would be of great interest to know the likelihood of developing ADRD later in their life among those breast cancer survivors. It is also imperative to examine whether common comorbid conditions (CVD, stroke, hypertension, and diabetes) affect the risk of ADRD when compared to those without these comorbidities. Therefore, this study aimed to determine the risk of AD and ADRD in association with cardiovascular disease, stroke, hypertension, and diabetes in a large cohort of women with breast cancer with up to 26 years of follow-up from1991to2016.Ourhypothesiswasthatinwomen with breast cancer, the risk of ADRD is significantly higher in patients who had a history of CVD, stroke, hypertension, and diabetes than those who did not.

## METHODS

### Data sources

This study used the de-identified Surveillance, Epidemiology, and End Results (SEER) and Medicare-linked database for breast cancer cases between 1991 and 2015 with follow-up from 1991 to 2016. The SEER program, supported by the National Cancer Institute (NCI), includes 17 population-based tumor registries in 8 states (Connecticut, Iowa, New Mexico, Utah, Hawaii, Kentucky, Louisiana, New Jersey), 7 metropolitan/rural areas (San Francisco/Oakland, Detroit, Atlanta, Seattle, Rural-Georgia, Los Angeles County, and San Jose-Monterey areas), Greater-California, and Greater-Georgia. The Medicare program provides payments for hospital, physician, and outpatient medical services for > 97% of persons aged ≥ 65 [12, 13]. The Committee for the Protection of Human Subjects at the University of Texas Health Science Center at Houston approved this study.

### Study population

**Fig. 1.**
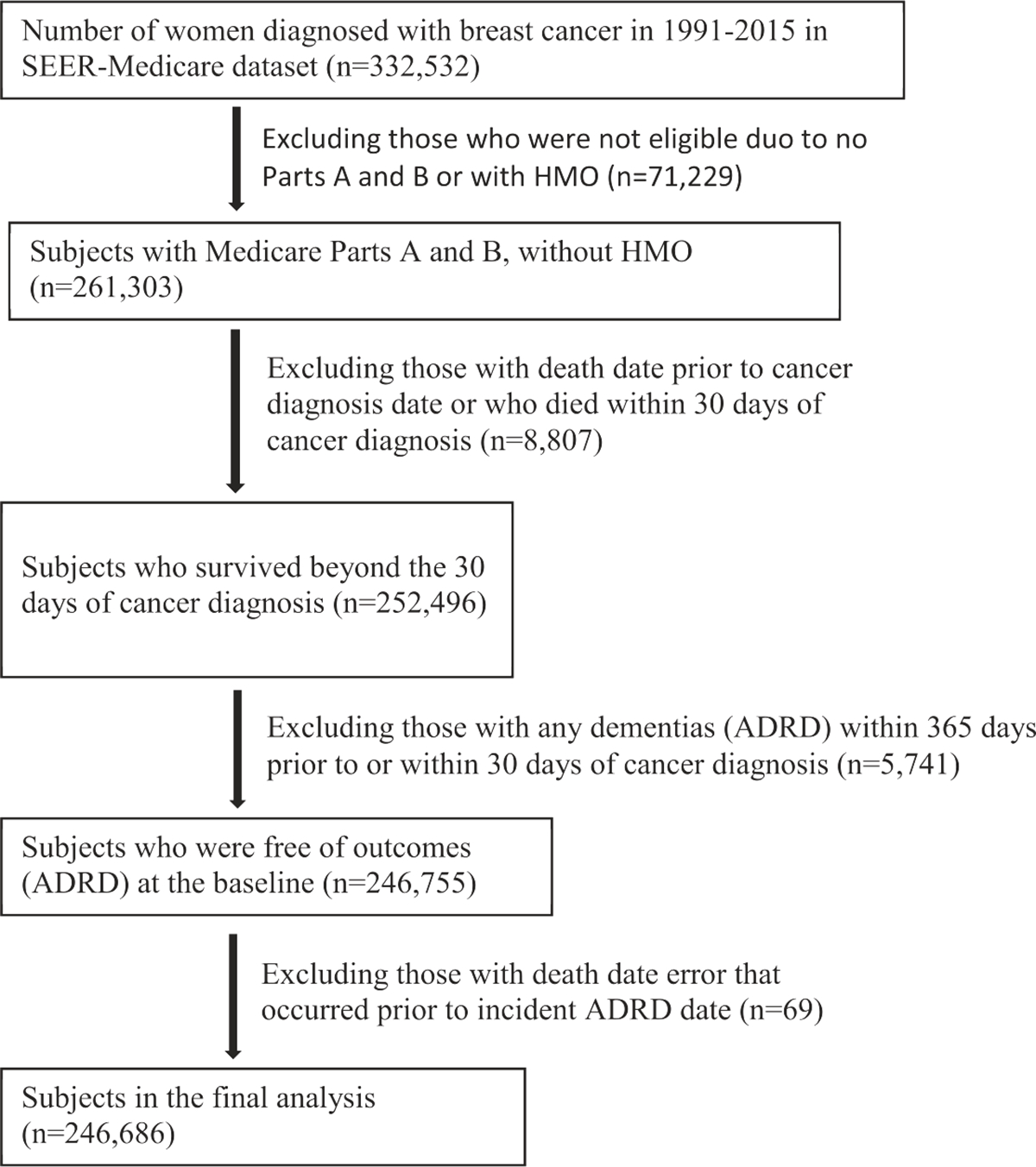
Flowchart of study participants included and excluded.

The study population consisted of 332,532 women who were diagnosed with breast cancer at age ≥ 65 years from 1991 through 2015 (Fig. 1). The study excluded those who did not have a full coverage of both Medicare Part A and Part B or who were enrolled with Health Maintenance Organizations from the date of diagnosis to the date of death or the date of last follow-up on December 31, 2016.The study excluded those who died within 30 days of breast cancer diagnosis and those who had a history of any dementias (ADRD) within 365 days prior to or within 30 days of cancer diagnosis. After exclusions, 246, 686 women with breast cancer who were free of ADRD at the baseline were left in the final analysis.

### Study variables

#### Main exposures

Main exposure variables are CVD (including myocardial infarction, congestive heart failure, or peripheral vascular disease), stroke, hypertension, and diabetes, which were defined as having an ICD-9 or ICD-10 diagnosis within 12 months prior to or 30 days after the date of cancer diagnosis (Supplementary Table 1).

#### Covariates

Covariates include socio-demographics (age, race/ethnicity, marital status), tumor factors (stage, size, grade, hormone receptor status, and receipt of chemotherapy and radiation therapy), comorbidity, year of diagnosis, and SEER areas. Comorbidities included chronic pulmonary disease, congestive tissue disease, ulcer disease, mild liver disease, hemiplegia, moderate or severe renal disease, leukemia, moderate or severe liver disease, and human immunodeficiency virus (HIV+) or acquired immune deficiency syndrome (AIDS). These co-existing conditions were identified through diagnoses or procedures in Medicare claims made within 1 year prior to and 30 days after the date of cancer diagnosis based on the NCI’s comorbidity coding program [14]. Each comorbid disease was weighted according to its severity and the sum of all scores was analyzed as 0, 1, and ≥ 2 [14].

#### Main outcomes

Main outcome variable was the incidence of ADRD. ADRD was defined if there was a diagnosis (ICD-9 or ICD-10) for any specific type of dementias (Supplementary Table 2). We used two approaches to define ADRD: 1) all diagnosis codes (from the first diagnosis code to the 12th diagnosis code available in Medicare claims) and 2) primary diagnosis code only (i.e., first diagnosis code). ADRD was classified into 6 specific types of dementias: AD, vascular dementia, dementia with Lewy bodies (Lewy), frontotemporal degeneration and dementias (FTD), mild cognitive impairment (MCI), and other dementias that were not included in the previous 5 categories.

#### Analysis

Differences in the distribution of baseline characteristics among breast cancer patients with CVD, stroke, hypertension, and diabetes were tested using the chi-square statistics. Cumulative incidence of ADRD is defined as the ratio of the number of cases with a new dementia over the number of population at risk who were free of dementia at the baseline when a breast cancer diagnosis was made. Incidence density is defined as the ratio of the number of cases with a new dementia over the total number of person-years by taking into consideration the different follow-up times of study participants. Person-years are calculated as the number of people multiplied by the number of years from the time of breast cancer diagnosis to the date of first dementia, or date of death, or date of last follow-up (December 31, 2016), whichever occurred first. Cox regression models were utilized to perform the time to event analysis to determine the risk of developing dementia by vascular diseases while adjusting for potential confounders. The proportionality assumption was assessed by checking whether the log-log Kaplan-Meier curves were parallel and did not intersect and also by adding an interaction term between exposures and time variables to the Cox models [15, 16]. Fine and Gray competing risk proportional hazards regression was performed accounting for death as a competing risk [16].

## RESULTS

Table 1 presents the distribution of baseline characteristics by the status of CVD, stroke, hypertension, and diabetes. Older women had a higher prevalence of CVD, stroke, and hypertension, but diabetes status was more evenly distributed by age. For example, the percentage of women who had CVD was 22.7% for age ≥ 85 and 14.7% for age 65–69, whereas the percentage of women who did not have CVD was 11.1%forage ≥ 85and28.4% for age 65–69. African American women and unmarried women appeared to have higher percentages of these vascular diseases. Women with advanced tumor stage, largersize, poorer grade, and hormone receptor negative tumors had a slightly higher prevalence of vascular diseases. As expected, patients who received chemotherapy or radiation therapy were less likely to have vascular diseases and those with higher comorbidity scores had a high percentage of these vascular diseases. There were some variations by years of diagnosis and by geographical areas in SEER.

Table 2 presents the cumulative incidence rates of AD, other specific types of dementias, and overall combined ADRD in a large cohort of women who were diagnosed with breast cancer and were free of ADRD at baseline (or at the time of cancer diagnosis) by the status of CVD, stroke, hypertension, diabetes, age, and other factors. The cumulative incidence of AD over the 26 years of follow-up varied from 10.7% to 13.6% by CVD, stroke, hypertension, and diabetes (second column in Table 2), whereas the cumulative incidence of AD as primary diagnosis only varied from 5.8% to 7.7% (in Supplementary Table 3). The cumulative incidence of any ADRD combined (last column in Table 2) was higher in those with CVD (40.75%) than those without CVD (31.32%), stroke (40.24%) than those without stroke (31.34%), hypertension (33.06%) than those without hypertension (30.47%), and diabetes (33.38%) than those without diabetes (31.77%).

Table 3 presents the incidence density of ADRD by taking into consideration differential follow-up times of women with breast cancer. The number of ADRD cases per 1,000 person-years were higher in those with CVD, stroke, hypertension, and diabetes than their counterparts. Tables 2 and 3 also present the cumulative incidence and incidence density rates of ADRD by age, race/ethnicity, tumor characteristics, comorbidity scores, chemotherapy, radiation therapy, and SEER areas. For example, the cumulative incidence of AD in women aged ≥ 65 with breast cancer and with up to 26 years of follow-up increased from 5.02% for age 65–69 to 9.42% forage70–74, 14.32% for age 75–79, and over 18% for age ≥ 80. The cumulative incidence of all ADRD increased from 17.67% for age 65–69 to 26.97% for age 70–74, 37.21% for age 75–79, and over 45% for age ≥ 80.

Table 4 presents the time to event hazard regression analyses after adjusting for potential confounders. Those with CVD (hazard ratio: 1.30, 95% CI: 1.27–1.33), stroke (1.50, 1.47–1.54), hypertension (1.08, 1.06–1.09), and diabetes (1.26, 1.24–1.29) had significantly higher risks of developing any ADRD combined (last column in Table 4). In terms of 6 specific types of dementias, CVD was associated with a significantly higher risk of AD, vascular dementia, and other dementias, was associated with a significantly decreased risk of MCI, but was not significantly associated with the risk of Lewy dementia and FTD. Stroke was associated with a significantly increased risk of AD, vascular dementia, Lewy dementia, MCI, and other dementias, but was not significantly associated with the risk of FTD. Hypertension was associated with a significantly lower risk of AD and vascular dementias and was not significantly associated with the risk of Lewy dementia and FTD but was associated with a significantly higher risk of MCI and other dementias. Diabetes was associated with a significantly higher risk of AD, Lewy dementia, MCI, and other dementias, but was not significantly associated with vascular dementia and FTD.

The risk of AD, non-AD dementias, and any ADRD all increased significantly withage. For example, the risk of AD was 1.94 (95% CI: 1.86–2.02) for age 70–74, 3.26 (3.13–3.40) for age 75–79, 5.29 (5.07–5.52) for age 80–84, and 7.42 (7.08–7.78) for age ≥ 85 as compared to those aged 65–69. As compared to white women, black women with breast cancer had a significantly higher risk of AD (1.21, 1.16–1.27) and any ADRD combined (1.16, 1.13–1.19), whereas Asians/Pacific Islanders had a significantly lower risk of AD (0.77, 0.71–0.83) and any ADRD combined (0.83, 0.79–0.86). Those unmarried women or those with unknown marital status had a significantly higher risk of AD and ADRD than those married women with breast cancer. The more advanced tumor stage, larger tumor size, poorer tumor grade, and negative hormone receptor status appeared to be associated with a small but significantly increased risk of AD and ADRD combined. Those who received chemotherapy and radiation therapy had a lower risk of AD and ADRD. The higher comorbidity scores were significantly associated with a higher risk of AD, MCI, other dementias, and ADRD combined. There were also some geographic variations in the risk of AD and ADRD, in which women from Connecticut, Detroit, Hawaii, and Kentucky had a significantly higher risk of AD and ADRD, while women from Iowa, Seattle, Utah, and Louisiana had a significantly lower risk of AD and ADRD as compared to women from California.

Table 5 presents the risk of different types of dementia in association with the number of vascular diseases (CVD, stroke, hypertension, and diabetes), which were classified into none of these diseases present or any 1, 2, 3, or 4 diseases present. As compared to women without any of these 4 diseases, those with any 2 or more of these diseases were significantly more likely to develop all types of dementias, including AD, vascular dementia, and total ADRD. Women with only one of these diseases present did not have a significantly increased risk of AD and vascular dementia but had a significantly higher risk of other types of dementia and total ADRD. There appears to be a dose-response relationship between the risk of ADRD and the number of vascular risk factors present. The point estimates for the risk of ADRD increased with the number of vascular diseases present. For example, the risk of ADRD increased from 1.14 (1.12–1.16) to 1.46 (1.43–1.49), 1.90 (1.84–1.96), and 2.47 (2.30–2.66) respectively for 1, 2, 3, and 4 vascular diseases present. Moreover, their 95% confidence intervals did not overlap,

The incidence rates and the adjusted risk of AD, non-AD dementias and ADRD combined as primary diagnosis only (Supplementary Tables 3–5) had the similar patterns by CVD, stroke, hypertension, diabetes, and other sociodemographic and tumor characteristics as the above results using any diagnosis code for ADRD. After taking into consideration death before ADRD as a competing risk in the Fine and Gray regression models, the risk of ADRD and other dementias were still significantly higher in women with CVD, stroke, hypertension, and diabetes, while the risk of AD remained significantly higher in women with stroke and significantly lower in women with hypertension, but the risk of AD was no longer significantly different in women with CVD or diabetes from those without CVD or diabetes. The risk of AD and ADRD by age, race/ethnicity, and other tumor factors remained similar without much changes. There were no significant interactions between chemotherapy and any of these four diseases (CVD, stroke, hypertension, and diabetes) on the risk of ADRD.

MCI can be a preclinical or prodromal stage of ADRD. It can also improve and stabilize. It is interesting and important to see the results on the risk of ADRD with and without including MCI. Hence, sensitivities analyses were performed by excluding MCI from the total ADRD. Supplementary Table 6 presents the 10-year and 26-year cumulative incidence rates of AD and ADRD by including MCI versus excluding MCI. As expected, the 26-year cumulative incidence of ADRD by excluding MCI (31.68%) was slightly lower than the incidence of ADRD that included MCI (32.14%). The 10-year cumulative incidence rate in women with breast cancer was 9.79% for AD, 27.88% for ADRD excluding MCI, and 28.31% for ADRD including MCI. The 10-year cumulative incidence rate of ADRD excluding MCI was higher in those with CVD than those without (38.71% versus 26.84%), and was the same for stroke (38.83% versus 26.79%), hypertension (29.84% versus 24.32%), and diabetes (31.11% versus 26.90%). The 10-year and 26-year cumulative incidence rates of AD and ADRD increased significantly with age and varied by other factors, which were similar to those found in Tables 2 and 3 above. Overall, excluding MCI did not materially change the study conclusions regarding the effects of vascular risk factors on the risk of ADRD in breast cancer survivors.

Supplementary Table 7 presents the adjusted 10-year and 26-year risk of AD and ADRD by including or excluding MCI. The 10-year and 26-year risk of both AD and ADRD regardless of including or excluding MCI was significantly higher in those with CVD, stroke, and diabetes than those without a history of these diseases, after adjusting for other confounding factors. For example, the 10-year risk of AD (1.32, 1.26–1.37), ADRD including MCI (1.51, 1.47–1.54), and ADRD excluding MCI (1.50, 1.47–1.54) was significantly higher in those with stroke than in those without stroke. Similarly, the 26-year risk of AD (1.32, 1.27–1.37), ADRD including MCI (1.50, 1.47–1.54), and ADRD excluding MCI (1.50, 1.47–1.53) was significantly higher in those with stroke than in those without stroke. Patients with hypertension had a significantly higher 10-year risk of total ADRD including MCI (1.07, 1.05–1.09) and ADRD excluding MCI (1.07, 1.05–1.08) and had a significantly higher 26-year risk of total ADRD including MCI (1.08, 1.06–1.09) and ADRD excluding MCI (1.07, 1.06–1.08) than those without hypertension. The 10-year risk (0.93, 0.91–0.96) and 26-year risk (0.96,0.93–0.98) of AD were slightly but significantly lower in those with hypertension than those without hypertension (Supplementary Table 7).

Supplementary Table 8 presents the risk of AD, other specific types of dementias, and total ADRD in association with different types of CVD (myocardial infarction, congestive heart failure, and peripheral vascular disease), stroke, hypertension, and diabetes in an entire cohort of women, and in stratified cohorts by age group (< 75 and ≥ 75 years). Myocardial infarction was not significantly associated with the risk of AD, vascular dementia, dementia with Lewy bodies, FTD, and MCI, but was associated with a significantly higher risk of other dementias and total ADRD. Congestive heart failure and peripheral vascular disease were significantly associated with an increased risk of AD, vascular dementia, other dementias and total ADRD; were associated with a significantly lower risk of MCI, but were not significantly associated with the risk of dementia with Lewy bodies and FTD. Furthermore, interactions between age (< 75 and ≥ 75 years) and vascular diseases (myocardial infarction, stroke, hypertension, and diabetes) on the risk of AD, vascular dementia, and total ADRD were statistically significant (*p*<0.05). Interactions between age (<75 and ≥ 75 years) and congestive heart failure or peripheral vascular disease were significant only for the risk of total ADRD but not significant for the risk of AD and vascular dementia.

In stratified analyses by age, myocardial infarction was significantly associated with an increased risk of AD (1.25, 1.10–1.43), vascular dementia (1.27, 1.00–1.62), other dementias (1.23, 1.14–1.32), and total ADRD (1.22, 1.14–1.31) in women aged < 75 years. In contrast, the association between myocardial infarction and the risk of different types of dementias and total ADRD in those aged 75 or older was similar to that of the entire cohort of women. Stroke was associated with a significantly higher risk of all types of dementias in both young (< 75 years) and older women (≥ 75 years). Hypertension, however, was associated with a significantly higher risk of AD and vascular dementia in women < 75 years but was associated with a significantly decreased risk of AD and vascular dementia in older women aged ≥ 75 years. Hypertension was associated with a significantly higher risk of MCI, other dementias and total ADRD, but was not associated with the risk of ementia with Lewy bodies and FTD in both age groups. Diabetes, on the other hand, was associated with a significantly higher risk of all types of dementias in both young(< 75 years) and older women (≥ 75 years), but there was no significant association between diabetes and the risk of FTD inwomen aged < 75years and the risk of vascular dementia in women aged ≥ 75 years.

## DISCUSSION

This study examined the long-term risk of AD, specific types of non-AD dementias, and all ADRD combined in association with CVD, stroke, hypertension, and diabetes in a large cohort of women diagnosed with breast cancer. The study found that CVD, stroke, hypertension, and diabetes were associated with a significantly higher risk of developing any ADRD combined. However, the risk of 6 specific types of dementias differed by CVD, stroke, hypertension, and diabetes. The CVD, stroke, and diabetes were associated with a significantly higher risk of AD, whereas hypertension was associated with a significantly lower risk of AD in this large cohort of older women aged ≥ 75 years. As expected, the risk of AD, non-AD dementias, and any ADRD allincreased significantly with age. Women aged 70–74, 75–79, 80–84, and ≥ 85 had 2-, 3-, 5-, and 7-fold higher risks of AD, respectively, as compared to those women aged 65–69 year.

The Framingham Heart Study [1, 7] reported that the estimated lifetime risk of any ADRD and only AD at age 65 was 24.6% and 21.1% for women, and 15.5% and 11.6% for men. Our study shows that the risk of any ADRD over 26 years of follow-up in older women with breast cancer at age ≥ 65 was 31.79% and the risk of only AD was 11.77%. The risk of ADRD in women with breast cancer at age ≥ 65 was higher than the lifetime risk for ADRD in Framingham women at age 65, but the risk of only AD in women with breast cancer at age ≥ 65 was lower than the lifetime risk for AD in women at age 65 from Framingham study [1, 7]. However, these comparisons might not be ideal between the Framingham community-based general population [7] and the current cohort of women who were diagnosed with breast cancer in SEER areas, but the information is critical for the estimated risk of ADRD and AD and should stimulate further investigations to see whether or why there is a gap among different populations. Several studies showed an inverse relationship between cancer and AD [17–19] or ADRD [20] and that the risk of AD dementia in persons with cancer could be reduced by 35% [18]. A recent systematic review and meta-analysis of 19 cohort studies and 3 case-control studies concluded that there was an inverse association between cancer and AD dementia, which was not likely attributable to diagnostic bias, competing risk bias, or inappropriate control for potential confounding factors [19].

Many previous studies also showed that the risk of ADRD was higher in those with vascular diseases. For example, patients with heart failure performed worse in neuropsychological testing than those healthy individuals and those with CVD, stroke, and hypertension also had a higher risk of dementia [8–11], whereas diabetes was shown to directly or indirectly affect the risk of ADRD as it is major risk factor for CVD, stroke, and hypertension. Numerous studies have documented that good management of cardiovascular risk factors (such as diabetes, obesity, smoking, and hypertension) is associated with the reduced risk of cognitive decline and may be associated with the reduced risk of dementia [8–11, 21, 22]. Interestingly, our study showed that hypertension in older women with breast cancer was associated with a significantly lower rather than higher risk of AD. It is unclear if this was due to breast cancer as discussed above for the association between cancer and ADRD, or due to older age in this cohort. The association between hypertension and ADRD became more complicated in mid-age versus older population. Many studies explored the complex relationship between hypertension and ADRD [23–25] and some found that hypertension at midlife is a risk factor for ADRD but hypertension at late life may have no effect or a weak protective effect for ADRD [23–25]. A number of studies, including a meta-analysis of longitudinal studies, showed that mid-life hypertension rather than late-life hypertension was a risk factor for AD [23–25]. Similarly, a comprehensive review concluded that the evidence on the association between treatment of hypertension in the elderly and the risk of dementia was inconclusive [26]. Although a recent large trial showed that intensive blood pressure control may be associated with a reduced risk of MCI [27], it is still unclear whether the risk of late stage of dementias such as AD will be decreased significantly. Our current study cannot answer this question directly, but it certainly raises an important research question for future research endeavor.

This study has a number of strengths. First, the study examined a large nationwide, population-based cohort of women with breast cancer aged ≥ 65 from the SEER areas, accounting for 26% of the U.S. population. Because ADRD takes a long time to develop, is relatively rare in young population, and is uncommon at the early stage of study follow-up, a very large study population is often required in order to generate meaningful information on the true incidence of ADRD and on potential risk factors associated with it. This large cohort of women, diagnosed with breast cancer in 1991–2015, has been followed up for up to 26 years, making this study unique and significant. Second, this study used the national comprehensive insurance program (Medicare) claims data to capture the incidence of AD and ADRD. Medicare is a federal health insurance program created in 1965 covering people aged ≥ 65, regardless of income, medical history, or health status. Medicare data are the lifelong medical records for these beneficiaries from the time of enrollment to the time of death regardless of where they seek medical care or services across the country, hence providing the most comprehensive lifetime history of medical records. Third, a large cohort of women with breast cancer were identified from the most authoritative cancer data source in NCI’s SEER program. SEER not only has a high complete case ascertainment rate, but also has high-quality information on tumor characteristics such as stage, size, and grade. Fourth, vascular diseases and comorbid conditions in women with breast cancer can be identified reliably from Medicare claims data which have been well validated for comorbidities in cancer patients [28–30].

This study also has a few limitations. First, because information on the history of CVD, stroke, hypertension, and diabetes relied on Medicare claims at baseline when breast cancer diagnosis was made, completeness and duration of these conditions as well as their treatment and control overtime are unknown, which could potentially affect the risk of ADRD. Second, although several studies [28–30] showed that Medicare claims data generally suggest a sensitivity of about 85% for overall dementia, there could be a certain degree of misclassification. It is possible that ADRD may be overestimated and AD only may be underestimated or misclassified in using Medicare claims data, which might explain the higher risk of ADRD and lower risk of AD only in our study cohort of women with breast cancer than the lifetime risk for ADRD and AD only in women from Framingham [1, 7]. Third, the study population only included elderly women with breast cancer aged ≥ 65. Therefore, the findings from this study may not be generalizable to younger patients. However, most ADRDs, hypertension, diabetes, and cancer occur in older persons (≥ 65 years), so the findings will still be generalizable to a larger population. Finally, the study cannot capture all relevant variables and there is a lack of information on factors such as smoking or family history, which might affect the risk of ADRD.

In conclusion, the study found that in a large cohort of women diagnosed with breast cancer at age ≥ 65 with up to 26 years of follow-up from 1991 to 2016, the overall patterns on the risk of ADRD and its association with vascular diseases and socio-demographic factors are consistent with the findings of previous studies, although the magnitude varied by the types of risk factors and types of ADRD. CVD, stroke, hypertension, and diabetes were associated with a significantly higher risk of developing any ADRD combined. The risk of 6 specific types of dementias differed by CVD, stroke, hypertension, and diabetes. CVD, stroke, and diabetes were associated with a significantly higher risk of AD, whereas hypertension was associated with a significantly lower risk of AD in those aged ≥ 75 years. The risk of ADRD significantly increased with age, was higher in unmarried women, and varied by race/ethnicity with a higher risk in black women and a lower risk in Asians/Pacific Islanders as compared to white women. Further studies may be needed to explore the effect of breast cancer on the risk of ADRD in a cohort of cancer patients when comparing it with women with similar background risk, but without breast cancer, and to examine whether and how the treatment for vascular diseases affect the risk of AD and non-AD dementias.

## Supplementary Material

Supplement file

## Figures and Tables

**Table 1:** Comparison of patient and tumor characteristics in women diagnosed with breast cancer by a history of cardiovascular diseases, stroke, hypertension, and diabetes in 1991–2015

Patient and Tumor Characteristics	Number of cases (column %) by cardiovascular diseases (CVD), stroke, hypertension, and diabetes							
	Cardiovascular diseases		Stroke		Hypertension		Diabetes	
	Yes	No	Yes	No	Yes	No	Yes	No

Median age (range)	78 (65,103)	74 (65,114)	77 (65,114)	74 (65,108)	75 (65,114)	72 (65,108)	74 (65,105)	74 (65,114)
Age (y)								
65–69	3,164 (14.7)	63,983 (28.4)	3,593 (16.2)	63,554 (28.3)	37,206 (23.4)	29,941 (34.1)	37,206 (25.2)	29,941 (27.8)
70–74	4,153 (19.2)	56,520 (25.1)	4,764 (21.4)	55,909 (24.9)	38,409 (24.2)	22,264 (25.4)	38,409 (25.6)	22,264 (24.3)
75–79	4,906 (22.7)	46,714 (20.8)	5,052 (22.7)	46,568 (20.8)	35,097 (22.1)	16,523 (18.8)	35,097 (22.2)	16,523 (20.5)
80–84	4,471 (20.7)	32,831 (14.6)	4,646 (20.9)	32,656 (14.6)	26,764 (16.8)	10,538 (12.0)	26,764 (15.8)	10,538 (14.9)
85 or older	4,901 (22.7)	25,043 (11.1)	4,177 (18.8)	25,767 (11.5)	21,495 (13.5)	8,449 (9.6)	21,495 (11.2)	8,449 (12.4)
Race/ethnicity								
Whites	18,213 (84.3)	194,939 (86.6)	18,857 (84.8)	194,295 (86.6)	134,309 (84.5)	78,843 (89.9)	45,135 (78.7)	168,017 (88.7)
Blacks	2,385 (11.0)	16,823 (7.5)	2,262 (10.2)	16,946 (7.6)	15,210 (9.6)	3,998 (4.6)	7,697 (13.4)	11,511 (6.1)
Asians/Pacific Islanders	536 (2.5)	9,218 (4.1)	834 (3.8)	8,920 (4.0)	6,806 (4.3)	2,948 (3.4)	3,295 (5.8)	6,459 (3.4)
Others	461 (2.1)	4,111 (1.8)	279 (1.3)	4,293 (1.9)	2,646 (1.7)	1,926 (2.2)	1,222 (2.1)	3,350 (1.8)
Marital status								
Married	6,694 (31.0)	100,779 (44.8)	6,694 (35.3)	100,779 (44.4)	65,891 (41.5)	41,582 (47.4)	22,456 (39.2)	85,017 (44.9)
Unmarried	13,786 (63.8)	113,271 (50.3)	13,786 (59.3)	113,271 (50.7)	85,165 (53.6)	41,892 (47.8)	31,829 (55.5)	95,228 (50.3)
Unknown	1,115 (5.2)	11,041 (4.9)	1,115 (5.4)	11,041 (4.9)	7,915 (5.0)	4,241 (4.8)	3,064 (5.3)	9,092 (4.8)
AJCC Tumor stage								
0 and I	9,463 (43.8)	125,083 (55.6)	11,439 (51.5)	123,107 (54.9)	87,153 (54.8)	47,393 (54.0)	29,688 (51.8)	104,858 (55.4)
II	6,893 (31.9)	62,117 (27.6)	6,181 (27.8)	62,829 (28.0)	44,620 (28.1)	24,390 (27.8)	16,861 (29.4)	52,149 (27.5)
III	1,979 (9.2)	15,891 (7.1)	1,878 (8.5)	15,992 (7.1)	11,875 (7.5)	5,995 (6.8)	4,831 (8.4)	13,039 (6.9)
IV	1,534 (7.1)	10,561 (4.7)	1,261 (5.7)	10,834 (4.8)	7,289 (4.6)	4,806 (5.5)	2,915 (5.1)	9,180 (4.9)
Unknown/Missing	1,726 (8.0)	11,439 (5.1)	1,473 (6.6)	11,692 (5.2)	8,034 (5.1)	5,131 (5.9)	3,054 (5.3)	10,111 (5.3)
Tumor size (cm)								
<1	3,227 (14.9)	49,006 (21.8)	4,148 (18.7)	48,085 (21.4)	33,410 (21.0)	18,823 (21.5)	10,883 (19.0)	41,350 (21.8)
1–<2	6,275 (29.1)	74,421 (33.1)	6,763 (30.4)	73,933 (32.9)	51,396 (32.3)	29,300 (33.4)	17,880 (31.2)	62,816 (33.2)
2–<3	4,150 (19.2)	38,160 (17.0)	3,990 (18.0)	38,320 (17.1)	27,791 (17.5)	14,519 (16.6)	10,407 (18.2)	31,903 (16.9)
3–<4	2,176 (10.1)	17,252 (7.7)	2,003 (9.0)	17,425 (7.8)	12,854 (8.1)	6,574 (7.5)	5,105 (8.9)	14,323 (7.6)
≥ 4	3,577 (16.6)	28,193 (12.5)	3,283 (14.8)	28,487 (12.7)	20,744 (13.1)	11,026 (12.6)	8,227 (14.4)	23,543 (12.4)
Missing	2,190 (10.1)	18,059 (8.0)	2,045 (9.2)	18,204 (8.1)	12,776 (8.0)	7,473 (8.5)	4,847 (8.5)	15,402 (8.1)
Tumor grade								
Well-differentiated	3,878 (18.0)	47,610 (21.2)	4,526 (20.4)	46,962 (20.9)	33,212 (20.9)	18,276 (20.8)	10,982 (19.2)	40,506 (21.4)
Moderately-differentiated	8,113 (37.6)	90,208 (40.1)	8,843 (39.8)	89,478 (39.9)	64,295 (40.4)	34,026 (38.8)	23,183 (40.4)	75,138 (39.7)
Poorly-differentiated	6,101 (28.3)	60,421 (26.8)	6,066 (27.3)	60,456 (26.9)	43,132 (27.1)	23,390 (26.7)	16,453 (28.7)	50,069 (26.4)
Unknown/Missing	3,503 (16.2)	26,852 (11.9)	2,797 (12.6)	27,558 (12.3)	18,332 (11.5)	12,023 (13.7)	6,731 (11.7)	23,624 (12.5)
Hormone receptor status								
Positive	15,082 (69.8)	169,925 (75.5)	16,689 (75.1)	168,318 (75.0)	121,003 (76.1)	64,004 (73.0)	43,628 (76.1)	141,379 (74.7)
Negative	2,831 (13.1)	28,875 (12.8)	2,964 (13.3)	28,742 (12.8)	20,384 (12.8)	11,322 (12.9)	7,450 (13.0)	24,256 (12.8)
Unknown	3,682 (17.1)	26,291 (11.7)	2,579 (11.6)	27,394 (12.2)	175,84 (11.1)	12,389 (14.1)	6,271 (10.9)	23,702 (12.5)
Chemotherapy (<12m)								
No	18,502 (85.7)	185,733 (82.5)	19,326 (86.9)	184,909 (82.4)	132,573 (83.4)	71,662 (81.7)	47,593 (83.0)	156,642 (82.7)
Yes	3,093 (14.3)	39,358 (17.5)	2,906 (13.1)	39,545 (17.6)	263,98 (16.6)	16,053 (18.3)	9,756 (17.0)	32,695 (17.3)
Radiotherapy (< 12 m)								
No	13,947 (64.6)	110,129 (48.9)	13,065 (58.8)	111,011 (49.5)	81,702 (51.4)	42,374 (48.3)	30,679 (53.5)	93,397 (49.3)
Yes	7,648 (35.4)	114,962 (51.1)	9,167 (41.2)	113,443 (50.5)	77,269 (48.6)	45,341 (51.7)	26,670 (46.5)	95,940 (50.7)
Comorbidity Scores								
0	12,494 (57.9)	177,591 (78.9)	13,470 (60.6)	176,615 (78.7)	114,840 (72.2)	75,245 (85.8)	38,965 (67.9)	151,120 (79.8)
1	6,466 (29.9)	39,925 (17.7)	6,166 (27.7)	40,225 (17.9)	35,388 (22.3)	11,003 (12.5)	13,612 (23.7)	32,779 (17.3)
≥2	2,635 (12.2)	7,575 (3.4)	2,596 (11.7)	7,614 (3.4)	8,743 (5.5)	1,467 (1.7)	4,772 (8.3)	5,438 (2.9)
Year of Diagnosis								
1992–1995	2,111 (9.8)	15,941 (7.1)	866 (3.9)	17,186 (7.7)	7,544 (4.8)	10,508 (12.0)	2,483 (4.3)	15,569 (8.2)
1996–1999	2,037 (9.4)	16,618 (7.4)	906 (4.1)	17,749 (7.9)	8,508 (5.4)	10,147 (11.6)	2,631 (4.6)	16,024 (8.5)
2000–2003	4,619 (21.4)	34,229 (15.2)	1,865 (8.4)	36,983 (16.5)	21,049 (13.2)	17,799 (20.3)	6,394 (11.2)	32,454 (17.1)
2004–2007	4,792 (22.2)	43,799 (19.5)	3,023 (13.6)	45,568 (20.3)	32,133 (20.2)	16,458 (18.8)	11,003 (19.2)	37,588 (19.9)
2008–2011	4,066 (18.8)	56,726 (25.2)	8,179 (36.8)	52,613 (23.4)	45,367 (28.5)	15,425 (17.6)	17,079 (29.8)	43,713 (23.1)
2012–2017	3,970 (18.4)	57,778 (25.7)	7,393 (33.3)	54,355 (24.2)	44,370 (27.9)	17,378 (19.8)	17,759 (31.0)	43,989 (23.2)
SEER Areas								
Connecticut	1,642 (7.6)	16,005 (7.1)	1,354 (6.1)	16,293 (7.3)	11,102 (7.0)	6,545 (7.5)	3,620 (6.3)	14,027 (7.4)
Detroit	2,316 (10.7)	16,175 (7.2)	1,858 (8.4)	16,633 (7.4)	12,139 (7.6)	6,352 (7.2)	4,918 (8.6)	13,573 (7.2)
Hawaii	195 (0.9)	3,184 (1.4)	262 (1.2)	3,117 (1.4)	2,214 (1.4)	1,165 (1.3)	859 (1.5)	2,520 (1.3)
Iowa	1,527 (7.1)	15,972 (7.1)	1,147 (5.2)	16,352 (7.3)	10,245 (6.4)	7,254 (8.3)	3,325 (5.8)	14,174 (7.5)
New Mexico	472 (2.2)	5,582 (2.5)	390 (1.8)	5,664 (2.5)	3,310 (2.1)	2,744 (3.1)	1,089 (1.9)	4,965 (2.6)
Seattle	1,153 (5.3)	14,892 (6.6)	1,046 (4.7)	14,999 (6.7)	8,531 (5.4)	7,514 (8.6)	2,669 (4.7)	13,376 (7.1)
Utah	571 (2.6)	6,089 (2.7)	337 (1.5)	6,323 (2.8)	3,568 (2.2)	3,092 (3.5)	1,262 (2.2)	5,398 (2.9)
Georgia	1,592 (7.4)	20,120 (8.9)	2,205 (9.9)	19,507 (8.7)	15,512 (9.8)	6,200 (7.1)	5,615 (9.8)	16,097 (8.5)
Kentucky	1,525 (7.1)	13,377 (5.9)	1,425 (6.4)	13,477 (6.0)	10,955 (6.9)	3,947 (4.5)	3,967 (6.9)	10,935 (5.8)
Louisiana	1,352 (6.3)	11,803 (5.2)	1,634 (7.4)	11,521 (5.1)	9,860 (6.2)	3,295 (3.8)	3,715 (6.5)	9,440 (5.0)
New Jersey	3,116 (14.4)	29,171 (13)	3,793 (17.1)	28,494 (12.7)	23,831 (15.0)	8,456 (9.6)	9,504 (16.6)	22,783 (12.0)
California	6,134 (28.4)	72,721 (32.3)	6,781 (30.5)	72,074 (32.1)	47,704 (30.0)	31,151 (35.5)	16,806 (29.3)	62,049 (32.8)
Total	21,595	225,091	22,232	224,454	158,971	87,715	57,349	189,337

**Table 2:** Cumulative incidence of ADRD by a history of CVD, stroke, hypertension, and diabetes in women with breast cancer with up to 26 years of follow-up from 1991 to 2016

Characteristics	Cumulative incidence (row %) of ADRD						
	AD	Vascular	Lewy	FTD	MCI	Others	Total

Cardiovascular disease							
No	11.59	4.47	0.79	0.24	2.43	29.24	31.32
Yes	13.60	6.23	0.66	0.13	1.83	38.07	40.75
Stroke							
No	11.67	4.35	0.77	0.23	2.30	29.28	31.34
Yes	12.83	7.38	0.87	0.20	3.18	37.41	40.24
Hypertension							
No	12.25	4.36	0.81	0.24	2.22	28.43	30.47
Yes Diabetes	11.50	4.76	0.76	0.22	2.46	30.88	33.06
No	12.10	4.53	0.81	0.23	2.38	29.68	31.77
Yes	10.69	4.90	0.76	0.21	2.37	31.11	33.38
Age (years)							
65–69	5.02	2.14	0.47	0.20	1.68	16.18	17.67
70–74	9.42	3.74	0.79	0.24	2.38	25.04	26.97
75–79	14.32	5.66	0.99	0.28	2.95	34.88	37.21
80–84	18.63	7.13	1.09	0.26	2.96	42.83	45.50
85 or older	18.73	7.04	0.69	0.15	2.22	46.74	49.69
Race/ethnicity							
Whites	11.79	4.53	0.79	0.24	2.45	30.06	32.17
Blacks	12.54	5.86	0.65	0.16	1.94	31.39	33.65
Asians/Pacific Islanders	8.02	3.35	0.56	0.15	1.71	22.96	25.14
Others	15.66	6.36	0.98	0.22	2.08	37.01	39.30
Marital status							
Married	9.76	3.59	0.77	0.22	2.35	24.94	26.87
Unmarried	13.48	5.51	0.78	0.23	2.41	34.33	36.64
Unknown	11.75	4.47	0.78	0.25	2.20	29.67	31.75
AJCC Tumor stage							
0 or I	11.87	4.59	0.84	0.25	2.75	29.19	31.34
II	12.85	5.29	0.80	0.24	2.29	32.48	34.68
III	9.35	3.46	0.60	0.15	1.55	28.20	30.11
IV	4.32	1.61	0.19	0.04	0.65	19.76	21.10
Unknown/Missing	15.21	5.77	0.85	0.22	1.78	37.39	39.91
Tumor size (cm)							
<1	11.26	4.39	0.86	0.25	2.84	27.87	30.06
1–<2	12.61	4.90	0.83	0.26	2.68	31.03	33.16
2–<3	12.32	5.13	0.80	0.22	2.22	31.49	33.73
3–<4	12.11	4.48	0.73	0.18	1.81	32.12	34.13
≥4	10.08	4.02	0.58	0.17	1.76	28.43	30.44
Missing	10.90	4.09	0.66	0.18	1.83	28.86	30.89
Tumor grade							
Well-differentiated	12.14	4.69	0.91	0.26	2.88	30.15	32.22
Moderately-differentiated	11.71	4.56	0.78	0.24	2.43	29.58	31.71
Poorly-differentiated	10.69	4.25	0.69	0.20	2.11	28.78	30.91
Unknown/Missing	13.68	5.48	0.72	0.18	1.91	33.86	36.12
Hormone receptor status							
Positive	11.42	4.49	0.78	0.24	2.47	29.25	31.36
Negative	10.12	3.91	0.66	0.19	2.13	27.32	29.28
Unknown	15.69	6.15	0.86	0.22	2.05	37.57	40.01
Chemotherapy							
No	12.48	4.91	0.82	0.23	2.43	31.00	33.20
Yes	8.36	3.25	0.55	0.21	2.13	25.25	27.05
Radiotherapy							
No	13.42	5.28	0.82	0.22	2.17	33.76	36.05
Yes	10.10	3.96	0.74	0.24	2.59	26.22	28.18
Comorbidity Scores							
0	12.29	4.73	0.81	0.24	2.37	29.94	32.04
1	10.12	4.15	0.67	0.20	2.47	29.75	31.90
≥2	9.55	4.79	0.60	0.19	2.02	32.61	35.10
SEER Areas							
Connecticut	14.83	9.55	0.78	0.32	2.56	35.63	38.18
Detroit	17.78	7.55	0.86	0.24	2.04	38.91	41.16
Hawaii	12.70	7.04	0.56	0.12	2.93	31.58	34.18
Iowa	11.97	3.88Lulu	0.73	0.22	1.83	32.57	34.13
New Mexico	11.12	3.85	0.89	0.21	1.95	32.11	33.63
Seattle	9.63	4.13	0.50	0.17	1.65	24.49	26.63
Utah	9.61	3.62	0.60	0.12	1.65	27.82	29.65
Georgia	10.20	3.28	0.71	0.20	1.88	24.86	26.85
Kentucky	12.18	3.76	0.87	0.24	1.55	32.20	33.47
Louisiana	10.64	3.22	0.69	0.17	1.77	26.55	28.53
New Jersey	11.75	4.76	0.81	0.27	2.60	28.60	31.04
California	10.81	3.84	0.84	0.23	3.05	29.35	31.62
Total	11.77	4.62	0.78	0.23	2.38	30.01	32.14

ADRD, Alzheimer’s disease and related dementias; AD, Alzheimer’s disease; Vascular, vascular dementia; Lewy, dementia with Lewy bodies; FTD, frontotemporal degeneration and dementias; MCI, mild cognitive impairment; others, other dementias; total, any of above ADR).

**Table 3:** Incidence density of ADRD by a history of CVD, stroke, hypertension, and diabetes in women with breast cancer with up to 26 years of follow-up from 1991 to 2016

Characteristics	Incidence density of ADRD (number of ADRD cases per 1,000 person-years)						
	AD	Vascular	Lewy	FTD	MCI	Others	Total

Cardiovascular disease							
No	18.13	6.98	1.23	0.37	3.80	45.72	48.97
Yes	30.83	14.12	1.50	0.30	4.16	86.33	92.41
Stroke							
No	18.19	6.78	1.20	0.36	3.58	45.66	48.87
Yes	29.88	17.18	2.03	0.47	7.41	87.13	93.74
Hypertension							
No	17.07	6.08	1.13	0.33	3.09	39.62	42.46
Yes	20.20	8.36	1.33	0.39	4.33	54.23	58.06
Diabetes							
No	18.38	6.89	1.21	0.36	3.62	45.09	48.26
Yes	21.25	9.74	1.43	0.41	4.70	61.84	66.34
Age (years)							
65–69	6.93	2.96	0.65	0.27	2.32	22.35	24.41
70–74	13.57	5.38	1.13	0.34	3.42	36.05	38.83
75–79	22.63	8.94	1.56	0.44	4.66	55.12	58.8
80–84	36.05	13.81	2.11	0.51	5.73	82.90	88.08
85 or older	52.01	19.55	1.92	0.42	6.17	129.80	137.99
Race/ethnicity							
Whites	18.74	7.2	1.26	0.38	3.90	47.78	51.14
Blacks	24.20	11.3	1.26	0.30	3.74	60.57	64.94
Asians/Pacific Islanders	13.35	5.58	0.94	0.26	2.85	38.25	41.87
Others	20.04	8.15	1.26	0.28	2.66	47.36	50.3
Marital status							
Married	14.09	5.18	1.12	0.32	3.40	36.02	38.81
Unmarried	23.59	9.64	1.37	0.41	4.22	60.10	64.14
Unknown	22.06	8.39	1.47	0.46	4.12	55.71	59.62
AJCC Tumor stage							
0 and I	17.17	6.63	1.21	0.36	3.97	42.21	45.34
II	20.50	8.45	1.27	0.38	3.65	51.81	55.32
III	21.07	7.8	1.35	0.34	3.50	63.60	67.89
IV	19.96	7.44	0.88	0.19	3.01	91.21	97.39
Unknown/Missing	30.13	11.43	1.68	0.44	3.52	74.06	79.04
Tumor size (cm)							
<1	15.90	6.2	1.21	0.36	4.01	39.36	42.46
1–<2	18.10	7.03	1.19	0.37	3.85	44.54	47.6
2–<3	20.00	8.33	1.30	0.36	3.60	51.11	54.75
3–<4	23.19	8.58	1.39	0.34	3.46	61.51	65.35
≥4	22.72	9.06	1.31	0.39	3.96	64.04	68.57
Missing	22.18	8.32	1.35	0.36	3.72	58.72	62.86
Tumor grade							
Well-differentiated	18.10	7	1.35	0.38	4.30	44.94	48.03
Moderately-differentiated	18.46	7.2	1.23	0.38	3.84	46.64	49.99
Poorly-differentiated	18.55	7.38	1.20	0.35	3.66	49.95	53.64
Unknown/Missing	22.79	9.12	1.21	0.31	3.19	56.38	60.14
Hormone receptor status							
Positive	18.18	7.16	1.25	0.38	3.94	46.57	49.93
Negative	17.98	6.95	1.18	0.34	3.78	48.51	51.99
Unknown	24.20	9.48	1.33	0.34	3.15	57.95	61.71
Chemotherapy							
No	20.35	8	1.34	0.38	3.96	50.56	54.15
Yes	12.57	4.88	0.83	0.32	3.20	37.96	40.65
Radiotherapy							
No	24.23	9.53	1.47	0.39	3.91	60.96	65.1
Yes	14.61	5.72	1.07	0.35	3.74	37.93	40.77
Comorbidity Scores							
0	18.53	7.13	1.23	0.36	3.58	45.13	48.3
1	19.94	8.17	1.32	0.40	4.88	58.64	62.88
≥2	25.35	12.72	1.59	0.49	5.36	86.57	93.2
SEER Areas							
Connecticut	21.80	14.04	1.14	0.47	3.76	52.37	56.12
Detroit	26.06	11.07	1.26	0.35	2.99	57.03	60.33
Hawaii	17.50	9.71	0.78	0.16	4.04	43.53	47.12
Iowa	16.72	5.42	1.02	0.31	2.56	45.51	47.7
New Mexico	16.58	5.74	1.33	0.32	2.91	47.89	50.15
Seattle	14.07	6.04	0.73	0.25	2.41	35.78	38.91
Utah	14.26	5.37	0.89	0.18	2.45	41.29	44.01
Georgia	19.08	6.13	1.33	0.38	3.52	46.49	50.21
Kentucky	21.57	6.67	1.53	0.43	2.74	57.01	59.26
Louisiana	19.58	5.93	1.27	0.32	3.26	48.84	52.49
New Jersey	20.80	8.43	1.43	0.48	4.61	50.60	54.93
California	17.42	6.18	1.36	0.37	4.92	47.30	50.97
Total	18.92	7.43	1.25	0.37	3.82	48.24	51.66

ADRD, Alzheimer’s disease and related dementias; AD, Alzheimer’s disease; Vascular, vascular dementia; Lewy, dementia with Lewy bodies; FTD, frontotemporal degeneration and dementias; MCI, mild cognitive impairment; others, other dementias; total, any of above ADRD.

**Table 4:** Hazard ratio (95% CI) of developing ADRD by a history of CVD, stroke, hypertension, and diabetes in women with breast cancer with up to 26 years of follow-up from 1991 to 2016

Characteristics	Hazard ratio (95% CI)* of developing ADRD by CVD, stroke, hypertension, and diabetes						
	AD	Vacular	Lewy	FTD	MCI	Others	Total

Cardiovascular disease							
No	1.00 (reference)	1.00 (reference)	1.00 (reference)	1.00 (reference)	1.00 (reference)	1.00 (reference)	1.00 (reference)
Yes	1.21 (1.16,1.26)	1.76 (1.63,1.89)	0.98 (0.82,1.17)	0.71 (0.49,1.04)	0.85 (0.77,0.95)	1.29 (1.26,1.32)	1.30 (1.27,1.33)
Stroke							
No	1.00 (reference)	1.00 (reference)	1.00 (reference)	1.00 (reference)	1.00 (reference)	1.00 (reference)	1.00 (reference)
Yes	1.32 (1.27,1.37)	1.43 (1.31,1.55)	1.54 (1.32,1.79)	1.25 (0.91,1.72)	1.79 (1.65,1.95)	1.49 (1.46,1.53)	1.50 (1.47,1.54)
Hypertension							
No	1.00 (reference)	1.00 (reference)	1.00 (reference)	1.00 (reference)	1.00 (reference)	1.00 (reference)	1.00 (reference)
Yes	0.96 (0.93,0.98)	0.87 (0.83,0.91)	1.05 (0.95,1.16)	1.12 (0.93,1.35)	1.25 (1.18,1.33)	1.08 (1.07,1.1)	1.08 (1.06,1.09)
Diabetes							
No	1.00 (reference)	1.00 (reference)	1.00 (reference)	1.00 (reference)	1.00 (reference)	1.00 (reference)	1.00 (reference)
Yes	1.15 (1.11,1.18)	1.05 (0.98,1.12)	1.22 (1.09,1.37)	1.21 (0.98,1.5)	1.28 (1.2,1.36)	1.27 (1.24,1.29)	1.26 (1.24,1.29)
Age (years)							
65–69	1.00 (reference)	1.00 (reference)	1.00 (reference)	1.00 (reference)	1.00 (reference)	1.00 (reference)	1.00 (reference)
70–74	1.94 (1.86,2.02)	1.83 (1.69,1.98)	1.67 (1.45,1.92)	1.21 (0.96,1.54)	1.42 (1.32,1.54)	1.62 (1.58,1.66)	1.61 (1.57,1.64)
75–79	3.26 (3.13,3.40)	2.96 (2.73,3.20)	2.26 (1.96,2.60)	1.53 (1.20,1.95)	1.93 (1.78,2.09)	2.54 (2.48,2.60)	2.49 (2.43,2.55)
80–84	5.29 (5.07,5.52)	4.27 (3.93,4.65)	3.09 (2.64,3.60)	1.78 (1.35,2.34)	2.43 (2.22,2.65)	3.83 (3.73,3.93)	3.75 (3.65,3.84)
85 or older	7.42 (7.08,7.78)	5.42 (4.93,5.96)	2.96 (2.45,3.57)	1.56 (1.08,2.24)	2.81 (2.53,3.12)	5.63 (5.48,5.79)	5.50 (5.35,5.65)
Race/ethnicity							
Whites	1.00 (reference)	1.00 (reference)	1.00 (reference)	1.00 (reference)	1.00 (reference)	1.00 (reference)	1.00 (reference)
Blacks	1.21 (1.16,1.27)	1.51 (1.39,1.64)	0.94 (0.78,1.14)	0.73 (0.50,1.07)	0.93 (0.84,1.04)	1.16 (1.13,1.19)	1.16 (1.13,1.19)
Asians/Pacific Islanders	0.77 (0.71,0.83)	0.67 (0.57,0.79)	0.76 (0.57,1.01)	0.75 (0.44,1.29)	0.55 (0.47,0.65)	0.81 (0.77,0.84)	0.83 (0.79,0.86)
Others	1.05 (0.97,1.13)	1.22 (1.07,1.39)	0.89 (0.66,1.21)	0.70 (0.37,1.33)	0.52 (0.43,0.64)	0.98 (0.93,1.03)	0.97 (0.93,1.02)
Marital status							
Married	1.00 (reference)	1.00 (reference)	1.00 (reference)	1.00 (reference)	1.00 (reference)	1.00 (reference)	1.00 (reference)
Unmarried	1.16 (1.13,1.19)	1.32 (1.25,1.39)	0.99 (0.90,1.09)	1.18 (0.99,1.41)	1.05 (1,1.11)	1.21 (1.19,1.23)	1.20 (1.18,1.22)
Unknown	1.12 (1.06,1.19)	1.08 (0.96,1.22)	1.12 (0.90,1.40)	1.39 (0.94,2.05)	1.21 (1.06,1.38)	1.14 (1.10,1.19)	1.14 (1.10,1.18)
AJCC Tumor stage							
0 or I	1.00 (reference)	1.00 (reference)	1.00 (reference)	1.00 (reference)	1.00 (reference)	1.00 (reference)	1.00 (reference)
II	1.11 (1.07,1.15)	1.34 (1.25,1.44)	1.02 (0.89,1.18)	1.07 (0.83,1.39)	0.92 (0.85,1.00)	1.11 (1.09,1.14)	1.11 (1.09,1.13)
III	1.24 (1.17,1.31)	1.19 (1.04,1.36)	1.34 (1.06,1.69)	1.04 (0.66,1.64)	0.88 (0.76,1.02)	1.37 (1.32,1.42)	1.35 (1.31,1.40)
IV	1.13 (1.03,1.24)	1.29 (1.05,1.59)	1.01 (0.66,1.56)	0.71 (0.29,1.77)	0.88 (0.70,1.11)	1.81 (1.73,1.89)	1.75 (1.67,1.83)
Unknown/Missing	1.41 (1.32,1.50)	1.67 (1.46,1.91)	1.30 (1.01,1.68)	1.24 (0.76,2.03)	0.79 (0.67,0.93)	1.41 (1.36,1.47)	1.41 (1.36,1.47)
Tumor size (cm)							
<1	1.00 (reference)	1.00 (reference)	1.00 (reference)	1.00 (reference)	1.00 (reference)	1.00 (reference)	1.00 (reference)
1–<2	1.08 (1.05,1.12)	1.11 (1.04,1.18)	0.97 (0.86,1.09)	1.01 (0.81,1.26)	0.93 (0.87,0.99)	1.07 (1.05,1.09)	1.06 (1.04,1.08)
2–<3	1.06 (1.01,1.11)	1.08 (0.99,1.18)	1.06 (0.89,1.25)	0.96 (0.70,1.32)	0.91 (0.83,1.01)	1.06 (1.03,1.09)	1.05 (1.02,1.08)
3–<4	1.16 (1.10,1.23)	0.97 (0.87,1.09)	1.14 (0.91,1.43)	0.94 (0.61,1.44)	0.90 (0.79,1.04)	1.16 (1.12,1.20)	1.14 (1.11,1.18)
≥4	1.15 (1.10,1.22)	0.97 (0.87,1.08)	1.10 (0.90,1.36)	1.17 (0.80,1.70)	1.09 (0.97,1.22)	1.15 (1.12,1.19)	1.14 (1.11,1.18)
Missing	0.95 (0.89,1.01)	0.66 (0.57,0.76)	0.99 (0.77,1.27)	0.98 (0.61,1.56)	1.16 (1.01,1.33)	0.98 (0.94,1.02)	0.97 (0.93,1.01)
Tumor grade							
Well-differentiated	1.00 (reference)	1.00 (reference)	1.00 (reference)	1.00 (reference)	1.00 (reference)	1.00 (reference)	1.00 (reference)
Moderately-differentiated	0.99 (0.95,1.02)	1.02 (0.96,1.09)	0.92 (0.82,1.03)	1.00 (0.81,1.24)	0.92 (0.86,0.98)	0.98 (0.96,1.00)	0.98 (0.97,1.00)
Poorly-differentiated	1.01 (0.97,1.04)	1.07 (0.99,1.15)	0.93 (0.81,1.07)	0.97 (0.75,1.25)	0.91 (0.84,0.98)	1.03 (1.01,1.06)	1.04 (1.02,1.06)
Unknown/Missing	1.03 (0.99,1.08)	1.20 (1.11,1.30)	0.82 (0.69,0.97)	0.77 (0.55,1.06)	0.78 (0.7,0.86)	1.03 (1,1.06)	1.04 (1.01,1.06)
Hormone receptor status							
Positive	1.00 (reference)	1.00 (reference)	1.00 (reference)	1.00 (reference)	1.00 (reference)	1.00 (reference)	1.00 (reference)
Negative	1.04 (1.00,1.09)	0.99 (0.91,1.07)	1.05 (0.9,1.23)	0.95 (0.71,1.27)	1.04 (0.95,1.13)	1.05 (1.02,1.07)	1.04 (1.02,1.07)
Unknown	1.11 (1.07,1.15)	1.38 (1.30,1.47)	0.92 (0.8,1.05)	0.84 (0.64,1.10)	0.74 (0.68,0.81)	1.09 (1.06,1.11)	1.09 (1.06,1.11)
Chemotherapy							
No	1.00 (reference)	1.00 (reference)	1.00 (reference)	1.00 (reference)	1.00 (reference)	1.00 (reference)	1.00 (reference)
Yes Radiotherapy	0.83 (0.80,0.86)	0.90 (0.83,0.97)	0.71 (0.61,0.83)	0.93 (0.72,1.21)	1.03 (0.95,1.12)	0.93 (0.90,0.95)	0.92 (0.90,0.94)
No	1.00 (reference)	1.00 (reference)	1.00 (reference)	1.00 (reference)	1.00 (reference)	1.00 (reference)	1.00 (reference)
Yes	0.79 (0.77,0.81)	0.81 (0.77,0.85)	0.82 (0.74,0.90)	0.92 (0.77,1.09)	1.03 (0.97,1.08)	0.81 (0.80,0.82)	0.81 (0.80,0.82)
Comorbidity Scores							
0	1.00 (reference)	1.00 (reference)	1.00 (reference)	1.00 (reference)	1.00 (reference)	1.00 (reference)	1.00 (reference)
1	1.04 (1.01,1.08)	0.91 (0.85,0.98)	1.10 (0.97,1.24)	1.16 (0.92,1.46)	1.34 (1.25,1.43)	1.20 (1.18,1.22)	1.20 (1.17,1.22)
≥2	1.15 (1.07,1.22)	1.14 (0.99,1.31)	1.23 (0.95,1.60)	1.49 (0.93,2.39)	1.32 (1.14,1.52)	1.45 (1.40,1.50)	1.45 (1.4,1.5)
SEER Areas							
Connecticut	1.14 (1.09,1.19)	2.11 (1.96,2.27)	0.75 (0.62,0.90)	1.13 (0.83,1.52)	0.67 (0.6,0.74)	1.05 (1.02,1.08)	1.05 (1.02,1.08)
Detroit	1.38 (1.32,1.43)	1.68 (1.55,1.81)	0.83 (0.70,0.99)	0.86 (0.61,1.20)	0.53 (0.47,0.59)	1.13 (1.10,1.16)	1.11 (1.08,1.14)
Hawaii	1.24 (1.12,1.37)	1.98 (1.67,2.36)	0.61 (0.38,0.99)	0.48 (0.17,1.33)	1.09 (0.88,1.35)	1.15 (1.08,1.23)	1.15 (1.08,1.22)
Iowa	0.85 (0.81,0.89)	0.91 (0.82,1.00)	0.66 (0.55,0.80)	0.75 (0.53,1.07)	0.47 (0.42,0.53)	0.90 (0.87,0.92)	0.87 (0.84,0.89)
New Mexico	0.95 (0.88,1.03)	1.05 (0.91,1.23)	0.94 (0.71,1.25)	0.83 (0.47,1.47)	0.60 (0.50,0.72)	1.08 (1.03,1.13)	1.04 (1.00,1.09)
Seattle	0.82 (0.78,0.87)	0.97 (0.88,1.08)	0.52 (0.41,0.66)	0.63 (0.42,0.95)	0.47 (0.41,0.53)	0.77 (0.75,0.80)	0.78 (0.75,0.80)
Utah	0.85 (0.78,0.92)	0.99 (0.85,1.15)	0.66 (0.48,0.91)	0.47 (0.23,0.96)	0.50 (0.41,0.60)	0.92 (0.88,0.97)	0.91 (0.87,0.95)
Georgia	1.17 (1.11,1.22)	0.59 (0.52,0.67)	1.05 (0.87,1.25)	1.08 (0.77,1.51)	0.70 (0.63,0.78)	0.97 (0.95,1.00)	0.97 (0.94,1.00)
Kentucky	1.29 (1.23,1.36)	0.93 (0.82,1.05)	1.14 (0.94,1.38)	1.12 (0.78,1.61)	0.52 (0.46,0.60)	1.24 (1.20,1.28)	1.18 (1.15,1.22)
Louisiana	1.11 (1.05,1.18)	0.63 (0.54,0.73)	0.97 (0.77,1.21)	0.91 (0.59,1.41)	0.64 (0.56,0.74)	0.94 (0.91,0.97)	0.94 (0.90,0.97)
New Jersey	1.17 (1.13,1.22)	1.12 (1.04,1.22)	1.04 (0.90,1.21)	1.27 (0.98,1.65)	0.86 (0.79,0.93)	0.98 (0.95,1.00)	0.98 (0.96,1.01)
California	1.00 (reference)	1.00 (reference)	1.00 (reference)	1.00 (reference)	1.00 (reference)	1.00 (reference)	1.00 (reference)

ADRD, Alzheimer’s disease and related dementias; AD, Alzheimer’s disease; Vascular, vascular dementia; Lewy, dementia with Lewy bodies; FTD, frontotemporal degeneration and dementias; MCI, mild cognitive impairment; others, other dementias; total, any of above ADRD; CVD, cardiovascular diseases.

* Hazard ratios were adjusted for cardiovascular disease, stroke, hypertension, diabetes, age, race/ethnicity, marital status, tumor stage, tumor size, tumor grade, hormone receptor status, chemotherapy, radiotherapy, comorbidity scores, and SEER areas.

**Table 5:** Hazard ratio (95% CI) of developing ADRD by a history of combination of risk factors (CVD, stroke, hypertension, and diabetes) in women with breast cancer with up to 26 years of follow-up from 1991 to 2016

Characteristics	Hazard ratio (95% CI)* of developing ADRD by CVD, stroke, hypertension, and diabetes						
	AD	Vascular	Lewy	FTD	MCI	Others	Total

Status of 4 risk factors (cardiovascular disease, stroke, hypertension, and diabetes)							
None	1.00 (reference)	1.00 (reference)	1.00 (reference)	1.00 (reference)	1.00 (reference)	1.00 (reference)	1.00 (reference)
1 of 4 risk factors	1.00 (0.97,1.03)	1.02 (0.97,1.08)	1.12 (1.00,1.24)	1.25 (1.02,1.52)	1.24 (1.17,1.33)	1.14 (1.12,1.16)	1.14 (1.12,1.16)
2 of 4 risk factors	1.16 (1.12,1.20)	1.18 (1.11,1.27)	1.31 (1.14,1.49)	1.31 (1.02,1.68)	1.69 (1.57,1.82)	1.46 (1.43,1.49)	1.46 (1.43,1.49)
3 of 4 risk factors	1.40 (1.33,1.48)	1.39 (1.24,1.57)	1.41 (1.11,1.77)	1.10 (0.68,1.79)	1.93 (1.71,2.18)	1.91 (1.85,1.97)	1.90 (1.84,1.96)
4 of 4 risk factors	1.60 (1.40,1.84)	1.81 (1.34,2.44)	3.21 (2.08,4.95)	1.58 (0.50,5.00)	2.35 (1.74,3.18)	2.45 (2.28,2.64)	2.47 (2.30,2.66)
Age							
65–69	1.00 (reference)	1.00 (reference)	1.00 (reference)	1.00 (reference)	1.00 (reference)	1.00 (reference)	1.00 (reference)
70–74	1.94 (1.86,2.02)	1.82 (1.69,1.97)	1.67 (1.44,1.92)	1.21 (0.95,1.53)	1.42 (1.32,1.54)	1.62 (1.58,1.66)	1.60 (1.57,1.64)
75–79	3.26 (3.12,3.40)	2.96 (2.74,3.20)	2.25 (1.95,2.59)	1.51 (1.18,1.92)	1.93 (1.78,2.08)	2.53 (2.47,2.60)	2.49 (2.43,2.54)
80–84	5.29 (5.06,5.52)	4.29 (3.95,4.67)	3.08 (2.64,3.59)	1.75 (1.33,2.30)	2.43 (2.23,2.65)	3.83 (3.73,3.93)	3.74 (3.65,3.84)
85 or older	7.44 (7.10,7.80)	5.56 (5.06,6.11)	2.95 (2.44,3.56)	1.51 (1.05,2.17)	2.81 (2.53,3.11)	5.64 (5.49,5.80)	5.51 (5.37,5.66)
Race/ethnicity							
Whites	1.00 (reference)	1.00 (reference)	1.00 (reference)	1.00 (reference)	1.00 (reference)	1.00 (reference)	1.00 (reference)
Blacks	1.20 (1.15,1.26)	1.48 (1.36,1.61)	0.94 (0.78,1.13)	0.74 (0.5,1.08)	0.92 (0.83,1.03)	1.15 (1.11,1.18)	1.15 (1.12,1.18)
Asians/Pacific Islanders	0.76 (0.71,0.82)	0.65 (0.55,0.76)	0.76 (0.57,1.01)	0.76 (0.44,1.30)	0.55 (0.47,0.65)	0.80 (0.76,0.84)	0.82 (0.78,0.85)
Others	1.05 (0.97,1.13)	1.21 (1.06,1.38)	0.89 (0.66,1.21)	0.70 (0.37,1.33)	0.52 (0.42,0.64)	0.97 (0.93,1.02)	0.97 (0.93,1.02)
Marital status							
Married	1.00 (reference)	1.00 (reference)	1.00 (reference)	1.00 (reference)	1.00 (reference)	1.00 (reference)	1.00 (reference)
Unmarried	1.16 (1.13,1.19)	1.32 (1.26,1.39)	0.99 (0.90,1.09)	1.18 (0.99,1.41)	1.05 (0.99,1.11)	1.21 (1.19,1.23)	1.20 (1.18,1.22)
Unknown	1.12 (1.06,1.19)	1.08 (0.95,1.22)	1.13 (0.91,1.40)	1.40 (0.95,2.06)	1.22 (1.07,1.38)	1.14 (1.10,1.19)	1.14 (1.11,1.18)
AJCC Tumor stage							
0 or I	1.00 (reference)	1.00 (reference)	1.00 (reference)	1.00 (reference)	1.00 (reference)	1.00 (reference)	1.00 (reference)
II	1.11 (1.07,1.15)	1.35 (1.26,1.45)	1.02 (0.89,1.17)	1.07 (0.83,1.38)	0.91 (0.84,0.99)	1.11 (1.09,1.14)	1.11 (1.09,1.14)
III	1.24 (1.17,1.32)	1.20 (1.05,1.37)	1.33 (1.05,1.69)	1.04 (0.66,1.64)	0.88 (0.76,1.01)	1.37 (1.33,1.42)	1.36 (1.31,1.40)
IV	1.13 (1.03,1.24)	1.32 (1.07,1.62)	1.01 (0.66,1.56)	0.71 (0.29,1.77)	0.87 (0.69,1.10)	1.82 (1.74,1.90)	1.76 (1.68,1.83)
Unknown/Missing	1.41 (1.33,1.50)	1.70 (1.49,1.94)	1.29 (1.00,1.67)	1.23 (0.75,2.02)	0.79 (0.67,0.93)	1.42 (1.37,1.47)	1.42 (1.37,1.47)
Tumor size (cm)							
<1	1.00 (reference)	1.00 (reference)	1.00 (reference)	1.00 (reference)	1.00 (reference)	1.00 (reference)	1.00 (reference)
1–<2	1.08 (1.05,1.12)	1.11 (1.04,1.18)	0.97 (0.86,1.09)	1.01 (0.81,1.26)	0.93 (0.87,0.99)	1.07 (1.05,1.09)	1.06 (1.04,1.09)
2–<3	1.06 (1.01,1.11)	1.09 (1.00,1.19)	1.06 (0.89,1.25)	0.96 (0.70,1.32)	0.92 (0.83,1.01)	1.06 (1.03,1.09)	1.05 (1.02,1.08)
3–≤4	1.17 (1.10,1.23)	0.98 (0.87,1.10)	1.14 (0.91,1.43)	0.94 (0.61,1.45)	0.90 (0.79,1.04)	1.16 (1.12,1.20)	1.15 (1.11,1.19)
≥4	1.16 (1.10,1.22)	0.97 (0.87,1.08)	1.10 (0.90,1.36)	1.17 (0.80,1.70)	1.09 (0.97,1.22)	1.15 (1.12,1.19)	1.15 (1.11,1.18)
Missing	0.95 (0.89,1.01)	0.65 (0.57,0.75)	0.99 (0.77,1.27)	0.98 (0.61,1.57)	1.17 (1.01,1.34)	0.98 (0.94,1.02)	0.97 (0.93,1.01)
Tumor grade							
Well-differentiated	1.00 (reference)	1.00 (reference)	1.00 (reference)	1.00 (reference)	1.00 (reference)	1.00 (reference)	1.00 (reference)
Moderately-differentiated	0.99 (0.96,1.02)	1.02 (0.95,1.08)	0.92 (0.82,1.03)	1.00 (0.81,1.25)	0.92 (0.86,0.98)	0.98 (0.96,1)	0.98 (0.97,1.00)
Poorly-differentiated	1.01 (0.97,1.04)	1.06 (0.99,1.14)	0.94 (0.81,1.08)	0.97 (0.75,1.26)	0.91 (0.84,0.98)	1.03 (1.01,1.06)	1.04 (1.02,1.06)
Unknown/Missing	1.04 (1.00,1.08)	1.21 (1.12,1.31)	0.82 (0.69,0.97)	0.77 (0.55,1.06)	0.78 (0.70,0.86)	1.03 (1.01,1.06)	1.04 (1.01,1.06)
Hormone receptor status							
Positive	1.00 (reference)	1.00 (reference)	1.00 (reference)	1.00 (reference)	1.00 (reference)	1.00 (reference)	1.00 (reference)
Negative	1.05 (1.00,1.09)	1.00 (0.92,1.08)	1.05 (0.90,1.23)	0.95 (0.71,1.27)	1.04 (0.95,1.13)	1.05 (1.02,1.08)	1.05 (1.02,1.07)
Unknown	1.11 (1.08,1.15)	1.40 (1.32,1.49)	0.91 (0.80,1.05)	0.84 (0.64,1.10)	0.74 (0.68,0.81)	1.09 (1.07,1.11)	1.09 (1.07,1.11)
Chemotherapy							
No	1.00 (reference)	1.00 (reference)	1.00 (reference)	1.00 (reference)	1.00 (reference)	1.00 (reference)	1.00 (reference)
Yes	0.83 (0.80,0.86)	0.89 (0.83,0.96)	0.71 (0.61,0.83)	0.93 (0.72,1.20)	1.03 (0.95,1.11)	0.92 (0.90,0.94)	0.92 (0.90,0.94)
Radiotherapy							
No	1.00 (reference)	1.00 (reference)	1.00 (reference)	1.00 (reference)	1.00 (reference)	1.00 (reference)	1.00 (reference)
Yes	0.79 (0.77,0.81)	0.80 (0.77,0.84)	0.82 (0.74,0.9)	0.92 (0.77,1.09)	1.03 (0.98,1.09)	0.81 (0.79,0.82)	0.81 (0.80,0.82)
Comorbidity Scores							
0	1.00 (reference)	1.00 (reference)	1.00 (reference)	1.00 (reference)	1.00 (reference)	1.00 (reference)	1.00 (reference)
1	1.05 (1.01,1.08)	0.93 (0.87,1.00)	1.09 (0.97,1.24)	1.15 (0.92,1.44)	1.33 (1.25,1.43)	1.20 (1.18,1.23)	1.20 (1.18,1.22)
≥2	1.16 (1.09,1.24)	1.21 (1.05,1.40)	1.24 (0.96,1.61)	1.51 (0.94,2.41)	1.34 (1.16,1.54)	1.48 (1.42,1.53)	1.47 (1.42,1.53)

ADRD, Alzheimer’s disease and related dementias; AD, Alzheimer’s disease; Vascular, vascular dementia; Lewy, dementia with Lewy bodies; FTD, frontotemporal degeneration and dementias; MCI, mild cognitive impairment; others, other dementias; total, any of above ADRD; CVD, cardiovascular diseases.

* Hazard ratios were adjusted for cardiovascular disease, stroke, hypertension, diabetes, age, race/ethnicity, marital status, tumor stage, tumor size, tumor grade, hormone receptor status, chemotherapy, radiotherapy, comorbidity scores, and SEER areas.

## Data Availability

The National Cancer Institute’s SEER (Surveillance, Epidemiology, and End Results)-Medicare Data User Agreement (DUA) specifically requests that “You (the Investigators) will not permit others to use the data except for collaborators within your institution involved with the research as described in your proposal”. However, the SEER-Medicare linked data are available to researchers from the National Cancer Institute upon signing the DUA, having the study proposal approved, and paying the related costs, which is available in their website: https://healthcaredelivery.cancer.gov/seermedicare/. We plan to share the statistical models and statistical programs that we used to analyze these data upon request and to share study findings and related study resources. We also plan to make results and algorithms available for verification or replication by other researchers.
