# Supplementary material for: Risk of Developing Alzheimer’s Disease and Related Dementias in Association with Cardiovascular Disease, Stroke, Hypertension, and Diabetes in a Large Cohort of Women with Breast Cancer and with up to 26 Years of Follow-Up
Source: J Alzheimers Dis. Author manuscript; Available in PMC 2022 May 19. (PMC9117151; doi:10.3233/JAD-215657)
Supplement: Supplement file [file NIHMS1803870-supplement-Supplement_file.pdf]

Supplementary Material

Risk of Developing Alzheimer's Disease and Related Dementias in Association with Cardiovascular Disease, Stroke, Hypertension, and Diabetes in a Large Cohort of Women with Breast Cancer and with up to 26 Years of Follow-Up

Supplementary Table 1. ICD-9 and ICD-10 codes for cardiovascular diseases, stroke, hypertension, and diabetes*

Medical Conditions	ICD-9 codes	ICD-10 codes
**Cardiovascular diseases**		
Myocardial infarction	410.x, 412.x	I21.x, I22.x, I25.2
Congestive heart failure	398.91, 402.01, 402.11, 402.91, 404.01, 404.03, 404.11, 404.13, 404.91, 404.93,425.4 - 425.9, 428.x	I09.9, I11.0, I13.0, I13.2, I25.5, I42.0, I42.5 - I42.9, I43.x, I50.x, P29.0
Peripheral vascular disease	093.0, 437.3, 440.x, 441.x, 443.1 - 443.9, 447.1, 557.1, 557.9, V43.4	I70.x, I71.x, I73.1, I73.8, I73.9, I77.1, I79.0, I79.2, K55.1, K55.8, K55.9, Z95.8, Z95.9
**Cerebrovascular disease (stroke)**	362.34, 430.x - 438.x	G45.x, G46.x, H34.0, I60.x - I69.x
**Hypertension**		
Hypertension, uncomplicated	401.x	I10.x
Hypertension, complicated	402.x - 405.x	I11.x - I13.x, I15.x
**Diabetes:**		
Diabetes without chronic complication	250.0 - 250.3, 250.8, 250.9	E10.0, E10.1, E10.6, E10.8, E10.9, E11.0, E11.1, E11.6, E11.8, E11.9, E12.0, E12.1, E12.6, E12.8, E12.9, E13.0, E13.1, E13.6, E13.8, E13.9, E14.0, E14.1, E14.6, E14.8, E14.9
Diabetes with chronic complication	250.4 - 250.7	E10.2 - E10.5, E10.7, E11.2 - E11.5, E11.7, E12.2 - E12.5, E12.7, E13.2 - E13.5, E13.7, E14.2 - E14.5, E14.7

*Sources:

[1] National Cancer Institute. SEER-Medicare: Calculation of Comorbidity Weights. http://healthcaredelivery.cancer.gov/seermedicare/program/comorbidity.html, accessed November 17, 2020.

[2] Alessandro Gasparini. Comorbidity scores. 2020-01-08. https://cran.microsoft.com/web/packages/comorbidity/vignettes/comorbidityscores.html, accessed November 17, 2020.

[3] Glasheen WP, Cordier T, Gumpina R, Haugh G, Davis J, Renda A (2019) Charlson Comorbidity Index: ICD-9 Update and ICD-10 Translation. *Am Health Drug Benefits* **12**, 188-197.

Supplementary Table 2. ICD-9 and ICD-10 codes for Alzheimer's disease and related dementias (ADRD)

ICD-9	Description for ICD-9 codes	ICD-10	Description for ICD-10 codes
290.0	Senile dementia, uncomplicated	F03.90	Unspecified dementia without behavioral disturbance
290.1	Presenile dementia, uncomplicated	F03.90	Unspecified dementia without behavioral disturbance
290.2	Senile dementia with delusional or depressive features	F03.90	Unspecified dementia without behavioral disturbance
290.3	Senile dementia with delirium (acute confusional state)	F03.90	Unspecified dementia without behavioral disturbance
290.40	Vascular dementia	F01.50	Vascular dementia
290.41	Vascular dementia, with delirium	F01.51	Vascular dementia with behavioral disturbance
290.42	Vascular dementia, with delusions		
290.43	Vascular dementia, with depressed mood		
290.8	Other specified senile psychotic conditions	F03.90	Unspecified dementia without behavioral disturbance
290.9	Unspecified senile psychotic condition	F03.90	Unspecified dementia without behavioral disturbance
291.2	Alcohol-induced persisting dementia	F10.27	Alcohol dependence with alcohol-induced dementia
292.82	Drug-induced persisting dementia	F19.97	Other psychoactive substance use, unspecified with psychoactive substance-induced persisting dementia
294.10	Dementia in conditions classified elsewhere without behavioral disturbance	F02.80	Dementia in other diseases classified elsewhere without behavioral disturbance
294.11	Dementia in conditions classified elsewhere with behavioral disturbance	F02.81	Dementia in other diseases classified elsewhere with behavioral disturbance
294.20	Dementia, unspecified, without behavioral disturbance	F03.90	Unspecified dementia without behavioral disturbance
294.21	Dementia, unspecified, with behavioral disturbance	F03.91	Unspecified dementia with behavioral disturbance
294.8	Other persistent mental disorders due to conditions classified elsewhere	F06.0	Psychotic disorder with hallucinations due to known physiological condition
294.9	Unspecified persistent mental disorders due to conditions classified elsewhere	F06.8	Other specified mental disorders due to known physiological condition
331.0	Alzheimer's disease	G30	Alzheimer's disease
		G30.0	Alzheimer's disease with early onset
		G30.1	Alzheimer's disease with late onset
		G30.8	Other Alzheimer's disease
		G30.9	Alzheimer's disease, unspecified
331.11	Frontotemporal dementia, Pick's disease	G31.01	Frontotemporal dementia, Pick's disease
331.19	Other frontotemporal dementia	G31.09	Other frontotemporal dementia
331.2	Senile degeneration of brain	G31.1	Senile degeneration of brain, not elsewhere classified
331.7	Cerebral degeneration in dis. classified elsewhere	G94	Other disorders of brain in dis. classified elsewhere
331.82	Dementia with Lewy bodies	G31.83	Dementia with Lewy bodies
331.83	Mild cognitive impairment, so stated	G31.84	Mild cognitive impairment, so stated
331.89	Other cerebral degeneration	G31.89	Other specified degenerative dis. of nervous system
331.9	Cerebral degeneration, unspecified	G31.9	Degenerative disease of nervous system, unspecified
797	Senility without mention of psychosis	R41.81	Age-related cognitive decline

1) Alzheimer's disease (AD)

331.0	Alzheimer's disease	G30	Alzheimer's disease
		G30.0	Alzheimer's disease with early onset
		G30.1	Alzheimer's disease with late onset
		G30.8	Other Alzheimer's disease
		G30.9	Alzheimer's disease, unspecified

2) Vascular dementia

290.40	Vascular dementia	F01.50	Vascular dementia
290.41	Vascular dementia, with delirium	F01.51	Vascular dementia with behavioral disturbance
290.42	Vascular dementia, with delusions		
290.43	Vascular dementia, with depressed mood		

3) Dementia with Lewy bodies

331.82	Dementia with Lewy bodies	G31.83	Dementia with Lewy bodies

4) Frontotemporal degeneration and dementias;

331.11	Frontotemporal dementia, Pick's disease	G31.01	Frontotemporal dementia, Pick's disease
331.19	Other frontotemporal dementia	G31.09	Other frontotemporal dementia

5) Mild cognitive impairment;

331.83	Mild cognitive impairment, so stated	G31.84	Mild cognitive impairment, so stated

6) All other dementias unspecified or all other dementias that were not specified above in categories 1-5 (see their codes in the Table).

290.0	Senile dementia, uncomplicated	F03.90	Unspecified dementia without behavioral disturbance
290.1	Presenile dementia, uncomplicated	F03.90	Unspecified dementia without behavioral disturbance
290.2	Senile dementia with delusional or depressive features	F03.90	Unspecified dementia without behavioral disturbance
290.3	Senile dementia with delirium (acute confusional state)	F03.90	Unspecified dementia without behavioral disturbance
290.8	Other specified senile psychotic conditions	F03.90	Unspecified dementia without behavioral disturbance
290.9	Unspecified senile psychotic condition	F03.90	Unspecified dementia without behavioral disturbance
291.2	Alcohol-induced persisting dementia	F10.27	Alcohol dependence with alcohol-induced dementia
292.82	Drug-induced persisting dementia	F19.97	Other psychoactive substance use, unspecified with psychoactive substance-induced persisting dementia
294.10	Dementia in conditions classified elsewhere without behavioral disturbance	F02.80	Dementia in other diseases classified elsewhere without behavioral disturbance
294.11	Dementia in conditions classified elsewhere with behavioral disturbance	F02.81	Dementia in other diseases classified elsewhere with behavioral disturbance
294.20	Dementia, unspecified, without behavioral disturbance	F03.90	Unspecified dementia without behavioral disturbance
294.21	Dementia, unspecified, with behavioral disturbance	F03.91	Unspecified dementia with behavioral disturbance
294.8	Other persistent mental disorders due to conditions classified elsewhere	F06.0	Psychotic disorder with hallucinations due to known physiological condition
294.9	Unspecified persistent mental disorders due to conditions classified elsewhere	F06.8	Other specified mental disorders due to known physiological condition
331.2	Senile degeneration of brain	G31.1	Senile degeneration of brain, not elsewhere classified
331.7	Cerebral degeneration in dis. classified elsewhere	G94	Other disorders of brain in dis. classified elsewhere
331.89	Other cerebral degeneration	G31.89	Other specified degenerative dis. of nervous system
331.9	Cerebral degeneration, unspecified	G31.9	Degenerative disease of nervous system, unspecified
797	Senility without mention of psychosis	R41.81	Age-related cognitive decline

Outcomes ADRD Grouping:
1) Alzheimer's disease (AD)
2) Vascular dementia (Vascular)
3) Dementia with Lewy bodies (Lewy)
4) Frontotemporal degeneration and dementias (FTD)
5) Mild cognitive impairment (MCI)
6) Other dementias, not mentioned in categories 1-5
7) Total (any ADRD or any of the above 1-6)

Supplementary Table 3. Cumulative incidence of ADRD as primary diagnosis by a history of CVD, stroke, hypertension, and diabetes in women with breast cancer with up to 26 years of follow-up from 1991 to 2016

	Cumulative incidence of ADRD (%)						
**Characteristics**	**AD**	**Vascular**	**Lewy**	**FTD**	**MCI**	**Others**	**Total**
**Cardiovascular disease**							
No	7.05	3.58	0.27	0.13	1.13	16.52	30.44
Yes	7.29	7.22	0.22	0.05	0.80	20.18	54.21
**Stroke**							
No	7.08	3.5	0.27	0.13	1.07	16.58	30.44
Yes	6.92	8.51	0.27	0.10	1.42	19.45	54.17
**Hypertension**							
No	7.70	3.22	0.30	0.15	1.09	16.79	27.53
Yes	6.72	4.22	0.25	0.11	1.11	16.86	34.97
**Diabetes**							
No	7.44	3.59	0.28	0.13	1.14	17.02	30.48
Yes	5.85	4.77	0.23	0.09	0.99	16.24	38.14
**Age (y)**							
65-69	3.17	1.55	0.19	0.13	0.87	8.97	14.65
70-74	6.04	2.85	0.30	0.15	1.16	14.51	24.52
75-79	8.90	4.67	0.36	0.16	1.31	20.33	37.73
80-84	10.97	6.95	0.32	0.09	1.32	24.14	55.21
85 or older	9.89	9.55	0.19	0.05	0.91	24.06	79.47
**Race/ethnicity**							
Whites	7.11	3.72	0.28	0.13	1.14	16.97	31.79
Blacks	7.17	5.81	0.22	0.07	0.86	17.44	39.36
Asians/Pacific Islanders	4.58	2.49	0.23	0.13	0.79	11.08	22.71
Others	9.86	3.86	0.26	0.09	0.96	20.49	31.16
**Marital status**							
Married	6.14	2.65	0.29	0.13	1.14	14.23	24.38
Unmarried	7.88	4.97	0.25	0.12	1.08	19.09	39.26
Unknown	6.75	4.19	0.26	0.14	1.06	16.35	36.33
**AJCC Tumor stage**							
0 or I	7.39	3.41	0.31	0.13	1.33	16.86	28.8
II	7.57	4.39	0.26	0.15	1.03	18.24	34.28
III	4.90	3.92	0.22	0.07	0.55	14.15	37.57
IV	2.12	3.7	0.03	0.02	0.21	8.71	47.17
Unknown/Missing	8.61	5.55	0.21	0.09	0.79	20.33	47.45
**Tumor size (cm)**							
<1	7.04	3.19	0.31	0.13	1.42	16.14	27
1-<2	7.86	3.67	0.30	0.14	1.25	17.99	30.33
2-<3	7.32	4.12	0.26	0.13	1.05	17.66	33.64
3-<4	6.84	4.43	0.24	0.11	0.76	17.04	38.51
≥4	5.54	4.68	0.21	0.08	0.70	14.61	39.26
Missing	6.07	4.19	0.19	0.10	0.82	15.62	37.58
**Tumor grade**							

Well-differentiated	7.48	3.54	0.28	0.13	1.39	17.30	30.38
Moderately-differentiated	6.99	3.77	0.28	0.14	1.12	16.53	30.83
Poorly-differentiated	6.42	3.77	0.27	0.12	0.96	15.86	32.45
Unknown/Missing	8.03	4.51	0.23	0.10	0.90	19.18	37.46
**Hormone receptor status**							
Positive	6.86	3.67	0.28	0.13	1.15	16.37	30.82
Negative	6.08	3.46	0.26	0.10	0.93	15.09	31.59
Unknown	9.37	4.99	0.24	0.13	0.99	21.59	38.81
**Chemotherapy**							
No	7.46	4.1	0.28	0.12	1.12	17.38	33.5
Yes	5.20	2.53	0.23	0.15	1.03	14.22	24.9
**Radiotherapy**							
No	7.80	4.82	0.26	0.10	0.95	18.61	39.53
Yes	6.33	2.99	0.28	0.14	1.27	15.05	25.75
**Comorbidity Scores**							
0	7.52	3.68	0.29	0.13	1.12	17.10	30.4
1	5.70	4.18	0.23	0.10	1.13	15.88	36.86
≥2	4.96	5.9	0.17	0.09	0.81	16.33	51.54
**SEER Areas**							
Connecticut	9.94	9.45	0.37	0.19	1.31	23.74	40.2
Detroit	11.49	6.36	0.25	0.16	0.85	24.96	41.8
Hawaii	7.93	4.9	0.27	0.03	1.24	16.60	28.6
Iowa	7.18	2.72	0.29	0.05	0.72	19.91	30.8
New Mexico	6.52	2.98	0.25	0.12	0.76	18.65	31.09
Seattle	6.28	2.8	0.21	0.11	0.80	13.39	23.86
Utah	5.44	2.18	0.17	0.03	0.74	14.11	24.73
Georgia	5.84	2.76	0.25	0.12	1.03	13.44	30.32
Kentucky	7.09	3.4	0.30	0.13	0.64	17.66	35.64
Louisiana	5.97	2.55	0.20	0.09	0.74	12.80	29.07
New Jersey	6.93	4.37	0.27	0.14	1.27	16.41	34.82
California	6.24	2.73	0.28	0.13	1.42	15.13	29.4
Total	7.07	3.81	0.27	0.12	1.11	16.84	31.92

ADRD, Alzheimer's disease and related dementias; AD, Alzheimer's disease; Vascular, vascular dementia; Lewy, dementia with Lewy bodies; FTD, frontotemporal degeneration and dementias; MCI, mild cognitive impairment; others, other dementias; total, any of above ADRD.

Supplementary Table 4. Incidence density of ADRD as primary diagnosis by a history of CVD, stroke, hypertension, and diabetes in women with breast cancer with up to 26 years of follow-up from 1991 to 2016

	Incidence density of ADRD (number of ADRD cases per 1,000 person-years)						
**Characteristics**	**AD**	**Vascular**	**Lewy**	**FTD**	**MCI**	**Others**	**Total**
**Cardiovascular disease**							
No	11.02	0.02	0.43	0.20	1.77	25.83	29.79
Yes	16.53	0.03	0.49	0.12	1.81	45.75	52.25
**Stroke**							
No	11.05	0.02	0.42	0.20	1.68	25.85	29.79
Yes	16.13	0.03	0.63	0.23	3.30	45.32	52.20
**Hypertension**							
No	10.73	0.04	0.42	0.21	1.52	23.40	26.98
Yes	11.80	0.01	0.45	0.19	1.96	29.61	34.10
**Diabetes**							
No	11.30	0.03	0.43	0.20	1.73	25.85	29.84
Yes	11.62	0.01	0.46	0.19	1.96	32.28	36.98
**Age (y)**							
65-69	4.38	0.01	0.26	0.17	1.20	12.40	14.31
70-74	8.69	0.01	0.42	0.22	1.67	20.89	24.04
75-79	14.06	0.02	0.57	0.25	2.07	32.13	36.87
80-84	21.23	0.06	0.62	0.18	2.56	46.72	53.92
85 or older	27.46	0.07	0.52	0.14	2.52	66.82	77.25
**Race/ethnicity**							
Whites	11.31	0.03	0.44	0.21	1.82	26.97	31.07
Blacks	13.83	0.02	0.42	0.13	1.67	33.65	38.20
Asians/Pacific Islanders	7.63	0.00	0.38	0.22	1.31	18.46	22.20
Others	12.62	0.00	0.34	0.11	1.23	26.23	30.54
**Marital status**							
Married	8.87	0.01	0.42	0.19	1.64	20.55	23.87
Unmarried	13.80	0.04	0.45	0.20	1.89	33.42	38.30
Unknown	12.67	0.03	0.49	0.26	1.99	30.71	35.37
**AJCC Tumor stage**							
0 or I	10.70	0.02	0.45	0.19	1.92	24.39	28.20
II	12.07	0.03	0.41	0.23	1.64	29.09	33.40
III	11.04	0.03	0.50	0.15	1.24	31.91	36.48
IV	9.81	0.08	0.15	0.08	0.99	40.23	45.84
Unknown/Missing	17.05	0.02	0.42	0.18	1.56	40.26	46.35
**Tumor size (cm)**							
<1	9.95	0.02	0.44	0.19	2.00	22.79	26.45
1-<2	11.29	0.02	0.43	0.20	1.79	25.82	29.66
2-<3	11.88	0.03	0.42	0.20	1.70	28.67	32.82
3-<4	13.09	0.05	0.46	0.22	1.46	32.62	37.73
≥4	12.47	0.03	0.47	0.19	1.57	32.92	38.13

Missing	12.35	0.02	0.38	0.20	1.67	31.78	36.56
**Tumor grade**							
Well-differentiated	11.15	0.01	0.42	0.19	2.07	25.78	29.73
Moderately-differentiated	11.03	0.03	0.44	0.21	1.76	26.06	30.11
Poorly-differentiated	11.14	0.02	0.46	0.20	1.66	27.53	31.69
Unknown/Missing	13.36	0.04	0.38	0.16	1.50	31.94	36.52
**Hormone receptor status**							
Positive	10.93	0.02	0.44	0.20	1.84	26.06	30.09
Negative	10.80	0.01	0.45	0.17	1.65	26.80	30.96
Unknown	14.45	0.05	0.37	0.20	1.53	33.29	37.90
**Chemotherapy**							
No	12.16	0.03	0.45	0.19	1.83	28.35	32.71
Yes	7.82	0.02	0.35	0.22	1.55	21.37	24.38
**Radiotherapy**							
No	14.08	0.04	0.47	0.19	1.71	33.60	38.52
Yes	9.15	0.01	0.40	0.21	1.83	21.76	25.23
**Comorbidity Scores**							
0	11.33	0.03	0.43	0.20	1.68	25.77	29.72
1	11.24	0.01	0.45	0.20	2.22	31.31	36.00
≥2	13.16	0.03	0.44	0.23	2.16	43.35	49.51
**SEER Areas**							
Connecticut	14.61	0.13	0.54	0.27	1.92	34.89	38.62
Detroit	16.84	0.02	0.37	0.23	1.25	36.59	40.84
Hawaii	10.93	0.00	0.37	0.04	1.71	22.89	27.62
Iowa	10.03	0.00	0.41	0.06	1.01	27.82	30.28
New Mexico	9.73	0.00	0.37	0.17	1.13	27.81	30.57
Seattle	9.18	0.00	0.31	0.15	1.17	19.57	23.27
Utah	8.07	0.04	0.25	0.04	1.09	20.94	24.18
Georgia	10.91	0.01	0.47	0.22	1.93	25.14	29.67
Kentucky	12.55	0.00	0.53	0.23	1.14	31.26	35.04
Louisiana	10.99	0.00	0.36	0.17	1.36	23.55	28.53
New Jersey	12.26	0.00	0.48	0.25	2.24	29.03	33.87
California	10.06	0.03	0.45	0.21	2.29	24.39	28.82
**Total**	11.36	0.02	0.43	0.20	1.78	27.06	31.18

ADRD, Alzheimer's disease and related dementias; AD, Alzheimer's disease; Vascular, vascular dementia; Lewy, dementia with Lewy bodies; FTD, frontotemporal degeneration and dementias; MCI, mild cognitive impairment; others, other dementias; total, any of above ADRD.

Supplementary Table 5. Hazard ratio (95% CI) of developing ADRD as primary diagnosis by a history of CVD, stroke, hypertension, and diabetes in women with breast cancer with up to 26 years of follow-up from 1991 to 2016

	Hazard ratio (95% CI)** of developing ADRD by CVD, stroke, hypertension, and diabetes						
**Characteristics**	**AD**	**Vascular**	**Lewy**	**FTD**	**MCI**	**Others**	**Total**
**Cardiovascular disease**							
No	1.00 (reference)	1.00 (reference)	1.00 (reference)	1.00 (reference)	1.00 (reference)	1.00 (reference)	1.00 (reference)
Yes	1.14 (1.08,1.20)	1.78 (1.59,2.00)	1.04 (0.77,1.41)	0.57 (0.31,1.06)	0.86 (0.73,1.00)	1.26 (1.21,1.30)	1.26 (1.23,1.30)
**Stroke**							
No	1.00 (reference)	1.00 (reference)	1.00 (reference)	1.00 (reference)	1.00 (reference)	1.00 (reference)	1.00 (reference)
Yes	1.26 (1.19,1.33)	1.39 (1.21,1.60)	1.49 (1.13,1.96)	1.32 (0.84,2.07)	1.79 (1.59,2.03)	1.4 (1.36,1.45)	1.42 (1.38,1.46)
**Hypertension**							
No	1.00 (reference)	1.00 (reference)	1.00 (reference)	1.00 (reference)	1.00 (reference)	1.00 (reference)	1.00 (reference)
Yes	0.94 (0.91,0.97)	0.77 (0.71,0.83)	1.00 (0.85,1.19)	0.96 (0.75,1.22)	1.18 (1.09,1.29)	1.03 (1.01,1.05)	1.03 (1.00,1.05)
**Diabetes**							
No	1.00 (reference)	1.00 (reference)	1.00 (reference)	1.00 (reference)	1.00 (reference)	1.00 (reference)	1.00 (reference)
Yes	1.06 (1.02,1.10)	1.02 (0.92,1.13)	1.17 (0.96,1.43)	1.02 (0.75,1.39)	1.13 (1.02,1.24)	1.18 (1.15,1.21)	1.18 (1.15,1.20)
**Age (y)**							
65-69	1.00 (reference)	1.00 (reference)	1.00 (reference)	1.00 (reference)	1.00 (reference)	1.00 (reference)	1.00 (reference)
70-74	1.96 (1.86,2.07)	1.76 (1.57,1.99)	1.60 (1.27,2.01)	1.25 (0.92,1.68)	1.36 (1.21,1.51)	1.66 (1.61,1.72)	1.66 (1.61,1.71)
75-79	3.21 (3.05,3.38)	2.79 (2.47,3.13)	2.15 (1.71,2.71)	1.45 (1.06,1.98)	1.68 (1.5,1.88)	2.56 (2.47,2.64)	2.57 (2.49,2.65)
80-84	4.98 (4.71,5.26)	3.92 (3.45,4.46)	2.46 (1.89,3.20)	1.08 (0.71,1.63)	2.14 (1.88,2.42)	3.7 (3.58,3.83)	3.76 (3.64,3.88)
85 or older	6.48 (6.09,6.88)	4.69 (4.04,5.43)	2.31 (1.65,3.24)	0.96 (0.54,1.70)	2.33 (2.00,2.72)	5.04 (4.85,5.23)	5.12 (4.94,5.30)
**Race/ethnicity**							
Whites	1.00 (reference)	1.00 (reference)	1.00 (reference)	1.00 (reference)	1.00 (reference)	1.00 (reference)	1.00 (reference)
Blacks	1.18 (1.11,1.25)	1.53 (1.34,1.75)	0.98 (0.71,1.35)	0.57 (0.32,1.01)	0.93 (0.79,1.09)	1.16 (1.12,1.21)	1.15 (1.12,1.19)
Asians/Pacific Islanders	0.74 (0.67,0.82)	0.64 (0.49,0.83)	0.84 (0.53,1.34)	1.30 (0.73,2.32)	0.58 (0.46,0.74)	0.73 (0.69,0.78)	0.74 (0.70,0.79)
Others	1.10 (1.00,1.21)	1.06 (0.84,1.32)	0.68 (0.38,1.23)	0.53 (0.19,1.42)	0.55 (0.4,0.74)	0.97 (0.9,1.03)	0.97 (0.91,1.03)
**Marital status**							
Married	1.00 (reference)	1.00 (reference)	1.00 (reference)	1.00 (reference)	1.00 (reference)	1.00 (reference)	1.00 (reference)
Unmarried	1.12 (1.09,1.16)	1.35 (1.25,1.46)	0.93 (0.79,1.10)	1.12 (0.89,1.43)	1.02 (0.94,1.10)	1.19 (1.17,1.22)	1.18 (1.16,1.20)
Unknown	1.06 (0.99,1.15)	1.15 (0.95,1.39)	1.15 (0.79,1.67)	1.46 (0.87,2.44)	1.26 (1.05,1.51)	1.12 (1.07,1.17)	1.11 (1.07,1.16)
**AJCC Tumor stage**							
0 or I	1.00 (reference)	1.00 (reference)	1.00 (reference)	1.00 (reference)	1.00 (reference)	1.00 (reference)	1.00 (reference)
II	1.06 (1.02,1.11)	1.42 (1.27,1.58)	0.86 (0.67,1.09)	1.20 (0.85,1.68)	0.85 (0.76,0.96)	1.11 (1.07,1.14)	1.11 (1.08,1.14)
III	1.10 (1.02,1.19)	1.26 (1.02,1.56)	1.19 (0.81,1.77)	0.81 (0.42,1.59)	0.65 (0.51,0.82)	1.27 (1.21,1.33)	1.25 (1.19,1.30)
IV	1.04 (0.91,1.18)	1.30 (0.93,1.80)	0.47 (0.17,1.28)	0.51 (0.12,2.11)	0.61 (0.41,0.91)	1.57 (1.47,1.68)	1.51 (1.42,1.60)
Unknown/Missing	1.39 (1.28,1.51)	1.36 (1.1,1.69)	0.94 (0.58,1.52)	0.8 (0.38,1.67)	0.75 (0.59,0.96)	1.35 (1.28,1.42)	1.35 (1.29,1.42)
**Tumor size (cm)**							
<1	1.00 (reference)	1.00 (reference)	1.00 (reference)	1.00 (reference)	1.00 (reference)	1.00 (reference)	1.00 (reference)
1-<2	1.09 (1.05,1.14)	1.18 (1.06,1.30)	0.95 (0.78,1.16)	1.04 (0.77,1.41)	0.88 (0.80,0.97)	1.08 (1.05,1.11)	1.07 (1.05,1.10)
2-<3	1.06 (1.00,1.12)	1.05 (0.91,1.20)	1.02 (0.77,1.37)	0.98 (0.64,1.51)	0.94 (0.81,1.08)	1.05 (1.01,1.09)	1.04 (1.01,1.08)
3-<4	1.13 (1.05,1.22)	0.99 (0.83,1.19)	1.19 (0.81,1.75)	1.07 (0.61,1.89)	0.85 (0.69,1.05)	1.11 (1.06,1.17)	1.11 (1.06,1.16)
≥4	1.12 (1.05,1.20)	0.93 (0.79,1.11)	1.22 (0.86,1.72)	1.13 (0.67,1.91)	0.99 (0.83,1.18)	1.11 (1.06,1.16)	1.11 (1.07,1.16)
Missing	0.89 (0.82,0.97)	0.78 (0.63,0.98)	0.99 (0.63,1.54)	1.34 (0.72,2.49)	1.08 (0.88,1.33)	0.96 (0.92,1.02)	0.96 (0.92,1.01)

**Tumor grade**							
Well-differentiated	1.00 (reference)	1.00 (reference)	1.00 (reference)	1.00 (reference)	1.00 (reference)	1.00 (reference)	1.00 (reference)
Moderately-differentiated	0.97 (0.93,1.00)	1.09 (0.99,1.21)	1.08 (0.88,1.32)	1.13 (0.84,1.53)	0.89 (0.81,0.98)	0.96 (0.94,0.99)	0.97 (0.95,0.99)
Poorly-differentiated	1.00 (0.96,1.05)	1.11 (0.99,1.25)	1.17 (0.92,1.48)	1.10 (0.77,1.57)	0.89 (0.80,1.00)	1.01 (0.98,1.04)	1.02 (0.99,1.04)
Unknown/Missing	1.01 (0.95,1.06)	1.23 (1.09,1.39)	0.92 (0.68,1.24)	0.81 (0.51,1.28)	0.79 (0.69,0.92)	1.03 (0.99,1.06)	1.03 (1.00,1.06)
**Hormone receptor status**							
Positive	1.00 (reference)	1.00 (reference)	1.00 (reference)	1.00 (reference)	1.00 (reference)	1.00 (reference)	1.00 (reference)
Negative	1.05 (1.00,1.10)	0.90 (0.79,1.03)	1.04 (0.81,1.34)	0.83 (0.56,1.23)	0.96 (0.84,1.10)	1.05 (1.01,1.08)	1.04 (1.01,1.08)
Unknown	1.11 (1.06,1.16)	1.53 (1.39,1.68)	0.75 (0.58,0.96)	0.92 (0.64,1.31)	0.79 (0.69,0.89)	1.11 (1.08,1.14)	1.10 (1.07,1.13)
**Chemotherapy**							
No	1.00 (reference)	1.00 (reference)	1.00 (reference)	1.00 (reference)	1.00 (reference)	1.00 (reference)	1.00 (reference)
Yes	0.86 (0.82,0.91)	0.84 (0.75,0.94)	0.86 (0.67,1.09)	1.13 (0.82,1.55)	1.13 (1.01,1.27)	0.95 (0.92,0.98)	0.94 (0.91,0.96)
**Radiotherapy**							
No	1.00 (reference)	1.00 (reference)	1.00 (reference)	1.00 (reference)	1.00 (reference)	1.00 (reference)	1.00 (reference)
Yes	0.81 (0.79,0.84)	0.79 (0.73,0.85)	0.87 (0.74,1.02)	1.04 (0.82,1.32)	1.10 (1.02,1.19)	0.82 (0.81,0.84)	0.83 (0.81,0.84)
**Comorbidity Scores**							
0	1.00 (reference)	1.00 (reference)	1.00 (reference)	1.00 (reference)	1.00 (reference)	1.00 (reference)	1.00 (reference)
1	1.00 (0.96,1.05)	0.95 (0.85,1.06)	1.11 (0.9,1.37)	1.12 (0.82,1.54)	1.31 (1.19,1.45)	1.16 (1.13,1.19)	1.15 (1.12,1.17)
≥2	1.08 (0.99,1.19)	1.10 (0.87,1.39)	1.07 (0.65,1.75)	1.48 (0.75,2.92)	1.19 (0.95,1.48)	1.38 (1.31,1.45)	1.37 (1.31,1.44)
**SEER Areas**							
Connecticut	1.32 (1.25,1.39)	3.21 (2.88,3.57)	1.06 (0.80,1.40)	1.19 (0.80,1.77)	0.75 (0.65,0.86)	1.37 (1.32,1.41)	1.31 (1.27,1.36)
Detroit	1.54 (1.46,1.62)	2.32 (2.07,2.60)	0.73 (0.53,1.00)	1.03 (0.68,1.56)	0.48 (0.41,0.57)	1.40 (1.36,1.45)	1.34 (1.30,1.38)
Hawaii	1.30 (1.14,1.48)	2.35 (1.78,3.09)	0.84 (0.41,1.70)	0.16 (0.02,1.21)	0.97 (0.70,1.35)	1.18 (1.08,1.30)	1.23 (1.14,1.34)
Iowa	0.88 (0.83,0.94)	1.11 (0.96,1.29)	0.78 (0.57,1.06)	0.28 (0.14,0.58)	0.40 (0.34,0.49)	1.07 (1.03,1.11)	0.98 (0.94,1.01)
New Mexico	0.95 (0.86,1.06)	1.29 (1.03,1.62)	0.81 (0.48,1.36)	0.78 (0.36,1.68)	0.50 (0.37,0.67)	1.19 (1.12,1.26)	1.09 (1.03,1.16)
Seattle	0.92 (0.86,0.99)	1.08 (0.92,1.28)	0.64 (0.44,0.91)	0.68 (0.41,1.15)	0.49 (0.41,0.59)	0.82 (0.79,0.86)	0.83 (0.80,0.87)
Utah	0.82 (0.74,0.91)	1.00 (0.78,1.28)	0.53 (0.29,0.97)	0.20 (0.05,0.81)	0.47 (0.36,0.63)	0.9 (0.84,0.96)	0.87 (0.82,0.93)
Georgia	1.17 (1.10,1.25)	0.69 (0.56,0.85)	1.12 (0.83,1.51)	1.18 (0.77,1.83)	0.84 (0.73,0.97)	1.05 (1.00,1.09)	1.04 (1.00,1.08)
Kentucky	1.32 (1.23,1.41)	0.70 (0.55,0.89)	1.19 (0.86,1.65)	1.10 (0.67,1.80)	0.48 (0.39,0.59)	1.31 (1.26,1.37)	1.23 (1.18,1.28)
Louisiana	1.11 (1.03,1.20)	0.53 (0.41,0.70)	0.84 (0.56,1.27)	0.89 (0.49,1.63)	0.59 (0.48,0.73)	0.90 (0.85,0.94)	0.92 (0.88,0.96)
New Jersey	1.22 (1.16,1.28)	0.99 (0.86,1.15)	1.06 (0.82,1.36)	1.24 (0.87,1.77)	0.91 (0.81,1.03)	1.12 (1.09,1.16)	1.12 (1.08,1.15)
California	1.00 (reference)	1.00 (reference)	1.00 (reference)	1.00 (reference)	1.00 (reference)	1.00 (reference)	1.00 (reference)

ADRD, Alzheimer's disease and related dementias; AD, Alzheimer's disease; Vascular, vascular dementia; Lewy, dementia with Lewy bodies; FTD, frontotemporal degeneration and dementias; MCI, mild cognitive impairment; others, other dementias; total, any of above ADRD; CVD, cardiovascular diseases

*Hazard ratios were adjusted for cardiovascular disease, stroke, hypertension, diabetes, age, race/ethnicity, marital status, tumor stage, tumor size, tumor grade, hormone receptor status, chemotherapy, radiotherapy, comorbidity scores, and SEER areas.

Supplementary Table 6. The 10-year and 26-year cumulative incidence of ADRD by a history of CVD, stroke, hypertension, and diabetes in women with breast cancer with up to 26 years of follow-up from 1991 to 2016

	The 10-year and 26-year cumulative incidence (row %) of AD and ADRD					
**Characteristics**	**10-year incidence of AD**	**10-year incidence of ADRD including MCI**	**10-year incidence of ADRD excluding MCI**	**26-year incidence of AD**	**26-year incidence of ADRD including MCI**	**26-year incidence of ADRD excluding MCI**
**Cardiovascular disease**						
No	9.52	27.28	26.84	11.59	31.32	30.84
Yes	12.56	39.03	38.71	13.60	40.75	40.43
**Stroke**						
No	9.54	27.21	26.79	11.67	31.34	30.89
Yes	12.28	39.43	38.83	12.83	40.24	39.64
**Hypertension**						
No	9.29	24.67	24.32	12.25	30.47	30.05
Yes	10.06	30.32	29.84	11.50	33.06	32.58
**Diabetes**						
No	9.80	27.30	26.90	12.10	31.77	31.32
Yes	9.76	31.63	31.11	10.69	33.38	32.86
**Age (y)**						
65-69	3.20	13.54	13.17	5.02	17.67	17.24
70-74	6.72	21.86	21.41	9.42	26.97	26.46
75-79	11.85	32.62	32.10	14.32	37.21	36.66
80-84	17.00	42.93	42.47	18.63	45.50	45.05
85 or older	18.25	48.85	48.52	18.73	49.69	49.37
**Race/ethnicity**						
Whites	9.79	28.26	27.83	11.79	32.17	31.70
Blacks	10.99	31.21	30.73	12.54	33.65	33.16
Asians/Pacific Islanders	6.40	21.84	21.44	8.02	25.14	24.70
Others	11.90	32.22	31.98	15.66	39.30	39.06
**Marital status**						
Married	7.50	22.34	21.90	9.76	26.87	26.39
Unmarried	11.68	33.27	32.85	13.48	36.64	36.18
Unknown	10.27	29.20	28.76	11.75	31.75	31.31
**AJCC Tumor stage**						
0 or I	9.58	26.88	26.38	11.87	31.34	30.80

II	10.84	30.80	30.41	12.85	34.68	34.26
III	8.67	28.68	28.35	9.35	30.11	29.75
IV	4.13	20.81	20.59	4.32	21.10	20.88
Unknown/Missing	13.12	36.21	35.94	15.21	39.91	39.61
**Tumor size (cm)**						
<1	8.88	25.34	24.83	11.26	30.06	29.49
1-<2	10.10	28.37	27.88	12.61	33.16	32.64
2-<3	10.41	30.01	29.63	12.32	33.73	33.32
3-<4	10.71	31.42	31.07	12.11	34.13	33.78
≥4	9.26	28.87	28.48	10.08	30.44	30.04
Missing	9.53	28.31	28.05	10.90	30.89	30.60
**Tumor grade**						
Well-differentiated	10.03	28.16	27.65	12.14	32.22	31.68
Moderately-differentiated	9.84	28.07	27.62	11.71	31.71	31.23
Poorly-differentiated	9.09	27.68	27.28	10.69	30.91	30.48
Unknown/Missing	10.75	30.73	30.41	13.68	36.12	35.75
**Hormone receptor status**						
Positive	9.57	27.75	27.29	11.42	31.36	30.87
Negative	8.47	26.17	25.75	10.12	29.28	28.84
Unknown	12.56	34.03	33.76	15.69	40.01	39.67
**Chemotherapy**						
No	10.50	29.40	28.96	12.48	33.20	32.73
Yes	6.36	23.08	22.66	8.36	27.05	26.60
**Radiotherapy**						
No	11.68	32.75	32.35	13.42	36.05	35.64
Yes	7.87	23.82	23.35	10.10	28.18	27.67
**Comorbidity Scores**						
0	9.99	27.59	27.18	12.29	32.04	31.59
1	9.10	29.93	29.42	10.12	31.90	31.38
≥2	9.17	34.45	33.94	9.55	35.10	34.59
**SEER Areas**						
Connecticut	11.32	31.63	31.30	14.83	38.18	37.76
Detroit	13.50	34.12	33.81	17.78	41.16	40.79
Hawaii	8.79	26.84	26.34	12.70	34.18	33.65
Iowa	8.90	27.24	26.95	11.97	34.13	33.80
New Mexico	8.49	28.13	27.82	11.12	33.63	33.32

Seattle	8.46	24.39	23.90	9.63	26.63	26.11
Utah	7.49	24.73	24.47	9.61	29.65	29.37
Georgia	9.63	25.93	25.50	10.20	26.85	26.43
Kentucky	10.90	31.50	31.33	12.18	33.47	33.29
Louisiana	10.56	28.40	27.95	10.64	28.53	28.11
New Jersey	10.58	28.61	28.15	11.75	31.04	30.54
California	8.76	27.54	26.97	10.81	31.62	31.02
Total	9.79	28.31	27.88	11.77	32.14	31.68

ADRD, Alzheimer's disease and related dementias; AD, Alzheimer's disease; MCI, mild cognitive impairment

Supplementary Table 7. The 10-year and 26-year hazard ratio (95% CI) of developing ADRD by a history of CVD, stroke, hypertension, and diabetes in women with breast cancer with up to 26 years of follow-up from 1991 to 2016

	Hazard ratio (95% CI)* of developing AD and ADRD by CVD, stroke, hypertension and diabetes					
**Characteristics**	**10-year risk of AD**	**10-year risk of ADRD including MCI**	**10-year risk of ADRD excluding MCI**	**26-year risk of AD**	**26-year risk of ADRD including MCI**	**26-year risk of ADRD excluding MCI**
**Cardiovascular disease**						
No	1.00 (reference)	1.00 (reference)	1.00 (reference)	1.00 (reference)	1.00 (reference)	1.00 (reference)
Yes	1.21 (1.16,1.26)	1.31 (1.28,1.34)	1.32 (1.29,1.35)	1.21 (1.16,1.26)	1.30 (1.27,1.33)	1.31 (1.28,1.34)
**Stroke**						
No	1.00 (reference)	1.00 (reference)	1.00 (reference)	1.00 (reference)	1.00 (reference)	1.00 (reference)
Yes	1.32 (1.26,1.37)	1.51 (1.47,1.54)	1.50 (1.47,1.54)	1.32 (1.27,1.37)	1.50 (1.47,1.54)	1.50 (1.46,1.53)
**Hypertension**						
No	1.00 (reference)	1.00 (reference)	1.00 (reference)	1.00 (reference)	1.00 (reference)	1.00 (reference)
Yes	0.93 (0.91,0.96)	1.07 (1.05,1.09)	1.07 (1.05,1.08)	0.96 (0.93,0.98)	1.08 (1.06,1.09)	1.07 (1.06,1.09)
**Diabetes**						
No	1.00 (reference)	1.00 (reference)	1.00 (reference)	1.00 (reference)	1.00 (reference)	1.00 (reference)
Yes	1.14 (1.11,1.18)	1.26 (1.24,1.28)	1.26 (1.24,1.28)	1.15 (1.11,1.18)	1.26 (1.24,1.29)	1.26 (1.24,1.28)
**Age (y)**						
65-69	1.00 (reference)	1.00 (reference)	1.00 (reference)	1.00 (reference)	1.00 (reference)	1.00 (reference)
70-74	2.07 (1.97,2.18)	1.63 (1.59,1.67)	1.64 (1.60,1.69)	1.94 (1.86,2.02)	1.61 (1.57,1.64)	1.61 (1.58,1.65)
75-79	3.74 (3.56,3.93)	2.57 (2.50,2.64)	2.60 (2.53,2.67)	3.26 (3.13,3.40)	2.49 (2.43,2.55)	2.52 (2.46,2.58)
80-84	5.99 (5.70,6.30)	3.86 (3.76,3.96)	3.92 (3.82,4.03)	5.29 (5.07,5.52)	3.75 (3.65,3.84)	3.80 (3.71,3.90)
85 or older	8.32 (7.89,8.78)	5.63 (5.48,5.80)	5.74 (5.58,5.91)	7.42 (7.08,7.78)	5.50 (5.35,5.65)	5.60 (5.45,5.75)
**Race/ethnicity**						
Whites	1.00 (reference)	1.00 (reference)	1.00 (reference)	1.00 (reference)	1.00 (reference)	1.00 (reference)
Blacks	1.20 (1.15,1.26)	1.16 (1.13,1.20)	1.17 (1.13,1.20)	1.21 (1.16,1.27)	1.16 (1.13,1.19)	1.16 (1.13,1.19)
Asians/Pacific Islanders	0.78 (0.72,0.85)	0.84 (0.8,0.88)	0.84 (0.80,0.88)	0.77 (0.71,0.83)	0.83 (0.79,0.86)	0.83 (0.79,0.87)
Others	1.11 (1.02,1.21)	1.01 (0.96,1.07)	1.02 (0.97,1.08)	1.05 (0.97,1.13)	0.97 (0.93,1.02)	0.99 (0.94,1.03)
**Marital status**						
Married	1.00 (reference)	1.00 (reference)	1.00 (reference)	1.00 (reference)	1.00 (reference)	1.00 (reference)
Unmarried	1.17 (1.14,1.20)	1.22 (1.20,1.24)	1.22 (1.20,1.24)	1.16 (1.13,1.19)	1.20 (1.18,1.22)	1.20 (1.19,1.22)
Unknown	1.11 (1.04,1.18)	1.15 (1.10,1.19)	1.14 (1.10,1.19)	1.12 (1.06,1.19)	1.14 (1.10,1.18)	1.14 (1.10,1.18)
**AJCC Tumor stage**						
0 or I	1.00 (reference)	1.00 (reference)	1.00 (reference)	1.00 (reference)	1.00 (reference)	1.00 (reference)
II	1.13 (1.09,1.18)	1.14 (1.11,1.16)	1.14 (1.11,1.17)	1.11 (1.07,1.15)	1.11 (1.09,1.13)	1.12 (1.09,1.14)

III	1.26 (1.19,1.34)	1.37 (1.33,1.42)	1.39 (1.34,1.44)	1.24 (1.17,1.31)	1.35 (1.31,1.40)	1.36 (1.32,1.41)
IV	1.14 (1.03,1.25)	1.78 (1.70,1.86)	1.80 (1.72,1.89)	1.13 (1.03,1.24)	1.75 (1.67,1.83)	1.77 (1.69,1.85)
Unknown/Missing	1.44 (1.35,1.54)	1.46 (1.41,1.52)	1.47 (1.41,1.53)	1.41 (1.32,1.50)	1.41 (1.36,1.47)	1.42 (1.37,1.48)
**Tumor size (cm)**						
<1	1.00 (reference)	1.00 (reference)	1.00 (reference)	1.00 (reference)	1.00 (reference)	1.00 (reference)
1-<2	1.09 (1.05,1.13)	1.07 (1.05,1.09)	1.07 (1.05,1.10)	1.08 (1.05,1.12)	1.06 (1.04,1.08)	1.07 (1.04,1.09)
2-<3	1.07 (1.01,1.12)	1.05 (1.02,1.09)	1.06 (1.03,1.09)	1.06 (1.01,1.11)	1.05 (1.02,1.08)	1.05 (1.03,1.08)
3-<4	1.17 (1.10,1.25)	1.15 (1.11,1.19)	1.15 (1.11,1.19)	1.16 (1.10,1.23)	1.14 (1.11,1.18)	1.15 (1.11,1.19)
≥4	1.18 (1.11,1.25)	1.16 (1.12,1.20)	1.16 (1.12,1.20)	1.15 (1.10,1.22)	1.14 (1.11,1.18)	1.15 (1.11,1.18)
Missing	0.97 (0.91,1.03)	0.98 (0.94,1.02)	0.98 (0.95,1.02)	0.95 (0.89,1.01)	0.97 (0.93,1.01)	0.97 (0.94,1.01)
**Tumor grade**						
Well-differentiated	1.00 (reference)	1.00 (reference)	1.00 (reference)	1.00 (reference)	1.00 (reference)	1.00 (reference)
Moderately-differentiated	0.99 (0.96,1.02)	0.98 (0.96,1.00)	0.98 (0.96,1.01)	0.99 (0.95,1.02)	0.98 (0.97,1.00)	0.99 (0.97,1.00)
Poorly-differentiated	1.01 (0.97,1.05)	1.04 (1.02,1.06)	1.04 (1.02,1.07)	1.01 (0.97,1.04)	1.04 (1.02,1.06)	1.04 (1.02,1.07)
Unknown/Missing	1.01 (0.97,1.06)	1.02 (1.00,1.05)	1.03 (1.00,1.05)	1.03 (0.99,1.08)	1.04 (1.01,1.06)	1.04 (1.01,1.06)
**Hormone receptor status**						
Positive	1.00 (reference)	1.00 (reference)	1.00 (reference)	1.00 (reference)	1.00 (reference)	1.00 (reference)
Negative	1.05 (1.00,1.09)	1.05 (1.03,1.08)	1.05 (1.03,1.08)	1.04 (1.00,1.09)	1.04 (1.02,1.07)	1.04 (1.02,1.07)
Unknown	1.13 (1.09,1.17)	1.09 (1.07,1.12)	1.10 (1.08,1.13)	1.11 (1.07,1.15)	1.09 (1.06,1.11)	1.09 (1.07,1.12)
**Chemotherapy**						
No	1.00 (reference)	1.00 (reference)	1.00 (reference)	1.00 (reference)	1.00 (reference)	1.00 (reference)
Yes	0.80 (0.77,0.84)	0.92 (0.90,0.95)	0.92 (0.90,0.94)	0.83 (0.80,0.86)	0.92 (0.90,0.94)	0.92 (0.90,0.94)
**Radiotherapy**						
No	1.00 (reference)	1.00 (reference)	1.00 (reference)	1.00 (reference)	1.00 (reference)	1.00 (reference)
Yes	0.76 (0.74,0.78)	0.79 (0.78,0.80)	0.79 (0.78,0.80)	0.79 (0.77,0.81)	0.81 (0.80,0.82)	0.81 (0.79,0.82)
**Comorbidity Scores**						
0	1.00 (reference)	1.00 (reference)	1.00 (reference)	1.00 (reference)	1.00 (reference)	1.00 (reference)
1	1.03 (1.00,1.07)	1.19 (1.17,1.22)	1.19 (1.17,1.22)	1.04 (1.01,1.08)	1.20 (1.17,1.22)	1.19 (1.17,1.22)
≥2	1.13 (1.06,1.21)	1.44 (1.39,1.49)	1.44 (1.39,1.49)	1.15 (1.07,1.22)	1.45 (1.40,1.50)	1.45 (1.40,1.50)
**SEER Areas**						
Connecticut	1.14 (1.09,1.20)	1.04 (1.01,1.08)	1.06 (1.02,1.09)	1.14 (1.09,1.19)	1.05 (1.02,1.08)	1.06 (1.03,1.09)
Detroit	1.40 (1.34,1.47)	1.12 (1.09,1.15)	1.14 (1.10,1.17)	1.38 (1.32,1.43)	1.11 (1.08,1.14)	1.12 (1.09,1.15)
Hawaii	1.18 (1.05,1.34)	1.13 (1.05,1.21)	1.12 (1.05,1.21)	1.24 (1.12,1.37)	1.15 (1.08,1.22)	1.15 (1.08,1.23)
Iowa	0.85 (0.80,0.89)	0.85 (0.83,0.88)	0.86 (0.84,0.89)	0.85 (0.81,0.89)	0.87 (0.84,0.89)	0.88 (0.85,0.90)
New Mexico	0.95 (0.87,1.04)	1.04 (0.99,1.10)	1.06 (1.01,1.11)	0.95 (0.88,1.03)	1.04 (1.00,1.09)	1.06 (1.01,1.10)
Seattle	0.96 (0.91,1.02)	0.88 (0.85,0.92)	0.89 (0.86,0.92)	0.82 (0.78,0.87)	0.78 (0.75,0.80)	0.78 (0.75,0.81)

Utah	0.87 (0.80,0.95)	0.92 (0.88,0.97)	0.93 (0.89,0.98)	0.85 (0.78,0.92)	0.91 (0.87,0.95)	0.92 (0.88,0.97)
Georgia	1.29 (1.23,1.36)	1.04 (1.01,1.07)	1.05 (1.02,1.08)	1.17 (1.11,1.22)	0.97 (0.94,1.00)	0.98 (0.95,1.01)
Kentucky	1.34 (1.27,1.41)	1.22 (1.18,1.26)	1.24 (1.20,1.28)	1.29 (1.23,1.36)	1.18 (1.15,1.22)	1.20 (1.17,1.24)
Louisiana	1.29 (1.21,1.36)	1.04 (1.00,1.08)	1.05 (1.01,1.08)	1.11 (1.05,1.18)	0.94 (0.90,0.97)	0.94 (0.91,0.98)
New Jersey	1.22 (1.17,1.27)	0.99 (0.97,1.02)	1.00 (0.97,1.02)	1.17 (1.13,1.22)	0.98 (0.96,1.01)	0.99 (0.96,1.01)
California	1.00 (reference)	1.00 (reference)	1.00 (reference)	1.00 (reference)	1.00 (reference)	1.00 (reference)

ADRD, Alzheimer's disease and related dementias; AD, Alzheimer's disease; MCI, mild cognitive impairment; CVD, cardiovascular diseases
*****Hazard ratios were adjusted for cardiovascular disease, stroke, hypertension, diabetes, age, race/ethnicity, marital status, tumor stage, tumor size, tumor grade, hormone receptor status, chemotherapy, radiotherapy, comorbidity scores, and SEER areas.

Supplementary Table 8. Hazard ratio (95% CI) of developing ADRD by a history of types of CVD, stroke, hypertension, and diabetes in women with breast cancer with up to 26 years of follow-up from 1991 to 2016, stratified by age

	Hazard ratio (95% CI)* of developing ADRD by types of CVD, stroke, hypertension and diabetes						
**Characteristics**	**AD**	**Vascular**	**Lewy**	**FTD**	**MCI**	**Others**	**Total**
**Types of CVD**							
**Myocardial infarction**							
No	1.00 (reference)	1.00 (reference)	1.00 (reference)	1.00 (reference)	1.00 (reference)	1.00 (reference)	1.00 (reference)
Yes	1.05 (0.98,1.12)	1.04 (0.91,1.19)	0.88 (0.66,1.17)	1.05 (0.62,1.76)	1.04 (0.90,1.21)	1.14 (1.10,1.18)	1.12 (1.08,1.16)
**Congestive heart failure**							
No	1.00 (reference)	1.00 (reference)	1.00 (reference)	1.00 (reference)	1.00 (reference)	1.00 (reference)	1.00 (reference)
Yes	1.18 (1.12,1.25)	1.88 (1.71,2.06)	1.08 (0.85,1.36)	0.70 (0.41,1.20)	0.77 (0.66,0.90)	1.27 (1.23,1.31)	1.28 (1.24,1.32)
**Peripheral vascular disease**							
No	1.00 (reference)	1.00 (reference)	1.00 (reference)	1.00 (reference)	1.00 (reference)	1.00 (reference)	1.00 (reference)
Yes	1.29 (1.19,1.39)	1.80 (1.58,2.05)	0.95 (0.67,1.36)	0.34 (0.11,1.07)	0.74 (0.59,0.94)	1.29 (1.23,1.35)	1.30 (1.24,1.36)
**Stroke**							
No	1.00 (reference)	1.00 (reference)	1.00 (reference)	1.00 (reference)	1.00 (reference)	1.00 (reference)	1.00 (reference)
Yes	1.32 (1.27,1.37)	1.43 (1.32,1.56)	1.54 (1.32,1.80)	1.25 (0.91,1.71)	1.79 (1.65,1.94)	1.49 (1.46,1.53)	1.51 (1.47,1.54)
**Hypertension**							
No	1.00 (reference)	1.00 (reference)	1.00 (reference)	1.00 (reference)	1.00 (reference)	1.00 (reference)	1.00 (reference)
Yes	0.96 (0.93,0.98)	0.87 (0.83,0.92)	1.06 (0.95,1.17)	1.12 (0.93,1.35)	1.25 (1.18,1.32)	1.08 (1.07,1.10)	1.08 (1.06,1.10)
**Diabetes**							
No	1.00 (reference)	1.00 (reference)	1.00 (reference)	1.00 (reference)	1.00 (reference)	1.00 (reference)	1.00 (reference)
Yes	1.15 (1.12,1.18)	1.05 (0.99,1.12)	1.22 (1.09,1.37)	1.21 (0.98,1.49)	1.28 (1.20,1.36)	1.27 (1.24,1.29)	1.27 (1.24,1.29)
**Age (y)**							
65-69	1.00 (reference)	1.00 (reference)	1.00 (reference)	1.00 (reference)	1.00 (reference)	1.00 (reference)	1.00 (reference)
70-74	1.94 (1.86,2.02)	1.83 (1.69,1.98)	1.67 (1.45,1.92)	1.21 (0.96,1.54)	1.42 (1.32,1.54)	1.62 (1.58,1.66)	1.61 (1.57,1.64)
75-79	3.26 (3.13,3.40)	2.95 (2.73,3.19)	2.26 (1.95,2.60)	1.53 (1.20,1.95)	1.93 (1.78,2.09)	2.54 (2.48,2.60)	2.49 (2.43,2.55)
80-84	5.29 (5.07,5.52)	4.27 (3.92,4.64)	3.08 (2.64,3.60)	1.78 (1.35,2.35)	2.43 (2.23,2.65)	3.83 (3.73,3.93)	3.75 (3.66,3.84)
85 or older	7.42 (7.08,7.78)	5.39 (4.91,5.92)	2.95 (2.44,3.57)	1.56 (1.09,2.24)	2.82 (2.54,3.13)	5.63 (5.48,5.79)	5.50 (5.35,5.65)
**Stratified by age**							
**<75 y**							
**Myocardial infarction**							
No	1.00 (reference)	1.00 (reference)	1.00 (reference)	1.00 (reference)	1.00 (reference)	1.00 (reference)	1.00 (reference)
Yes	1.25 (1.10,1.43)	1.27 (1.00,1.62)	1.06 (0.65,1.73)	1.39 (0.65,3.00)	1.24 (0.96,1.59)	1.23 (1.14,1.32)	1.22 (1.14,1.31)
**Congestive heart failure**							
No	1.00 (reference)	1.00 (reference)	1.00 (reference)	1.00 (reference)	1.00 (reference)	1.00 (reference)	1.00 (reference)
Yes	1.29 (1.14,1.46)	1.80 (1.47,2.21)	1.11 (0.69,1.77)	0.66 (0.24,1.79)	0.81 (0.61,1.08)	1.52 (1.42,1.62)	1.48 (1.40,1.58)
**Peripheral vascular disease**							
No	1.00 (reference)	1.00 (reference)	1.00 (reference)	1.00 (reference)	1.00 (reference)	1.00 (reference)	1.00 (reference)
Yes	1.31 (1.11,1.56)	1.68 (1.28,2.21)	0.79 (0.37,1.67)	0.34 (0.05,2.42)	0.96 (0.66,1.40)	1.54 (1.40,1.68)	1.49 (1.36,1.63)
**Stroke**							
No	1.00 (reference)	1.00 (reference)	1.00 (reference)	1.00 (reference)	1.00 (reference)	1.00 (reference)	1.00 (reference)
Yes	1.69 (1.47,1.74)	2.10 (1.80,2.46)	1.80 (1.36,2.38)	1.68 (1.03,2.72)	1.90 (1.64,2.20)	1.73 (1.65,1.81)	1.74 (1.66,1.82)
**Hypertension**							

No	1.00 (reference)	1.00 (reference)	1.00 (reference)	1.00 (reference)	1.00 (reference)	1.00 (reference)	1.00 (reference)
Yes	1.14 (1.09,1.19)	1.10 (1.02,1.20)	1.04 (0.90,1.21)	1.14 (0.88,1.47)	1.20 (1.10,1.30)	1.21 (1.18,1.24)	1.20 (1.17,1.23)
**Diabetes**							
No	1.00 (reference)	1.00 (reference)	1.00 (reference)	1.00 (reference)	1.00 (reference)	1.00 (reference)	1.00 (reference)
Yes	1.24 (1.18,1.31)	1.22 (1.10,1.36)	1.27 (1.06,1.53)	0.90 (0.64,1.26)	1.29 (1.16,1.42)	1.37 (1.33,1.41)	1.37 (1.33,1.41)
**>=75 y**							
**Myocardial infarction**							
No	1.00 (reference)	1.00 (reference)	1.00 (reference)	1.00 (reference)	1.00 (reference)	1.00 (reference)	1.00 (reference)
Yes	1.01 (0.94,1.09)	0.98 (0.83,1.15)	0.8 (0.56,1.15)	0.85 (0.42,1.74)	0.96 (0.80,1.16)	1.13 (1.08,1.18)	1.11 (1.06,1.16)
**Congestive heart failure**							
No	1.00 (reference)	1.00 (reference)	1.00 (reference)	1.00 (reference)	1.00 (reference)	1.00 (reference)	1.00 (reference)
Yes	1.22 (1.15,1.29)	1.98 (1.78,2.19)	1.08 (0.83,1.41)	0.72 (0.38,1.37)	0.78 (0.65,0.93)	1.28 (1.24,1.33)	1.30 (1.25,1.34)
**Peripheral vascular disease**							
No	1.00 (reference)	1.00 (reference)	1.00 (reference)	1.00 (reference)	1.00 (reference)	1.00 (reference)	1.00 (reference)
Yes	1.33 (1.22,1.44)	1.87 (1.62,2.17)	1.03 (0.69,1.55)	0.35 (0.09,1.41)	0.67 (0.50,0.90)	1.25 (1.19,1.33)	1.28 (1.21,1.35)
**Stroke**							
No	1.00 (reference)	1.00 (reference)	1.00 (reference)	1.00 (reference)	1.00 (reference)	1.00 (reference)	1.00 (reference)
Yes	1.31 (1.25,1.37)	1.31 (1.18,1.45)	1.46 (1.22,1.76)	1.04 (0.69,1.58)	1.76 (1.59,1.94)	1.48 (1.44,1.52)	1.49 (1.45,1.53)
**Hypertension**							
No	1.00 (reference)	1.00 (reference)	1.00 (reference)	1.00 (reference)	1.00 (reference)	1.00 (reference)	1.00 (reference)
Yes	0.93 (0.90,0.96)	0.79 (0.75,0.84)	1.11 (0.97,1.26)	1.13 (0.86,1.47)	1.35 (1.24,1.46)	1.06 (1.04,1.09)	1.06 (1.04,1.08)
**Diabetes**							
No	1.00 (reference)	1.00 (reference)	1.00 (reference)	1.00 (reference)	1.00 (reference)	1.00 (reference)	1.00 (reference)
Yes	1.06 (1.02,1.10)	0.92 (0.85,1.00)	1.17 (1.01,1.35)	1.49 (1.13,1.96)	1.25 (1.15,1.36)	1.16 (1.13,1.18)	1.16 (1.13,1.18)

ADRD, Alzheimer's disease and related dementias; AD, Alzheimer's disease; Vascular, vascular dementia; Lewy bodies, dementia with Lewy bodies; FTD, frontotemporal degeneration and dementias; MCI, mild cognitive impairment; others, other dementias; total, any of above ADRD; CVD, cardiovascular diseases.

*Hazard ratios were adjusted for types of cardiovascular disease (myocardial infarction, congestive heart failure, peripheral vascular disease), stroke, hypertension, diabetes, age, race/ethnicity, marital status, tumor stage, tumor size, tumor grade, hormone receptor status, chemotherapy, radiotherapy, comorbidity scores, and SEER areas.
